# Mechanistic insights into the (3 + 2) cycloaddition of azomethine ylide with dimethyl acetylenedicarboxylate *via* bond evolution theory

**DOI:** 10.1039/d5ra04992j

**Published:** 2025-08-21

**Authors:** Mohamed Chellegui, Raghad Mowafak Al-Mokhtar, Raad Nasrullah Salih, Lakhdar Benhamed, Sofiane Benmetir, Jesus Vicente de Julián-Ortiz, Haydar A. Mohammad-Salim, Ali Ben Ahmed

**Affiliations:** a Laboratory of Organic Chemistry (LR17ES08), Faculty of Sciences, University of Sfax Sfax 3038 Tunisia; b Namur Institute of Structured Matter, University of Namur Rue de Bruxelles, 61 Namur B-5000 Belgium; c Department of Chemistry, College of Science, University of Duhok Duhok 42001 Kurdistan Region Iraq; d Nursing Department, Bardarash Technical Institute, Akre University for Applied Science Duhok 42001 Kurdistan Region Iraq; e Laboratory of Applied Thermodynamics and Molecular Modelling (LAT2M), Department of Chemistry, Faculty of Science, University of Tlemcen PB 119 Tlemcen 13000 Algeria; f Department of Physical Chemistry, Faculty of Pharmacy, University of Valencia Av. Vicente Andrés Estellés s/n Valencia 46100 Spain; g Process and Environmental Engineering Laboratory (LIPE), Faculty of Chemistry, University of Science and Technology of Oran Mohamed BOUDIAF P.O. Box 1503, El Mnaouer Oran 31000 Algeria; h Department of Chemistry, Faculty of Science, University of Zakho Zakho 42002 Kurdistan Region Iraq; i TCCG Lab, Scientific Research Center, University of Zakho Zakho 42002 Kurdistan Region Iraq; j Laboratory of Applied Physics, Department of Physics, Faculty of Sciences of Sfax, University of Sfax Sfax Tunisia; k Department of Biomedical, Higher Institute of Biotechnology of Sfax, University of Sfax Sfax Tunisia ali.benahmed@isbs.usf.tn

## Abstract

The mechanistic pathway of the (3 + 2) cycloaddition (32CA) between azomethine ylide 1 and dimethyl acetylenedicarboxylate 2, affording 4-isoxazoline derivatives, was elucidated *via* Density Functional Theory (DFT) calculations employing the B3LYP-D3 functional and the 6-311++G(d,p) basis set in 1,4-dioxane. Reactivity insights derived from Conceptual DFT (CDFT) demonstrated that compound 1 behaves as an ambiphilic species with significant nucleophilic and electrophilic tendencies, whereas compound 2 functions predominantly as an electrophile. These electronic features reveal a marked polarity in the cycloaddition and align with a forward electron density flux (FEDF) governing the reaction process. Natural Population Analysis (NPA) and Parr functions identified the C3 carbon of 1 as the most nucleophilic center and the C4/C5 carbons of 2 as the most electrophilic, suggesting initial C3–C4 bond formation. Thermodynamic analysis showed the *endo* cycloadduct to be more stable, while kinetic data favored the *exo* pathway, suggesting a kinetically controlled mechanism that ultimately leads to thermodynamically preferred *endo*-selectivity. The geometries of the transition states revealed asynchronous bond formation, with the *exo* pathway exhibiting a higher degree of asynchronicity. Global Electron Density Transfer (GEDT) values confirmed the moderately to distinctly polar nature of the pathways. Finally, detailed Electronic Localization Function (ELF) topological analysis and Bonding Evolution Theory (BET) elucidated the asynchronous, multistage mechanism involving six structural stability domains (SSDs), characterizing the formation of new bonds through a sequence of topological catastrophes. Non-Covalent Interaction (NCI) analysis provided visual and quantitative evidence of attractive and repulsive intermolecular forces influencing the TS geometries.

## Abstract 2

DFT at the B3LYP-D3/6-311++G(d,p) level reveals a polar, asynchronous, multistage 32CA mechanism between azomethine ylide and dimethyl acetylenedicarboxylate, with kinetic preference for formation of the *exo* cycloadduct.

## Graphical abstract

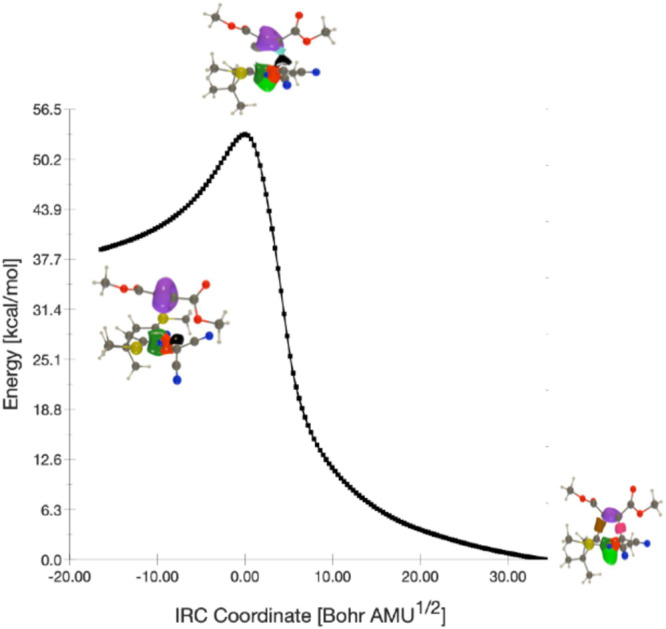

## 1. Introduction

**Scheme 1:**
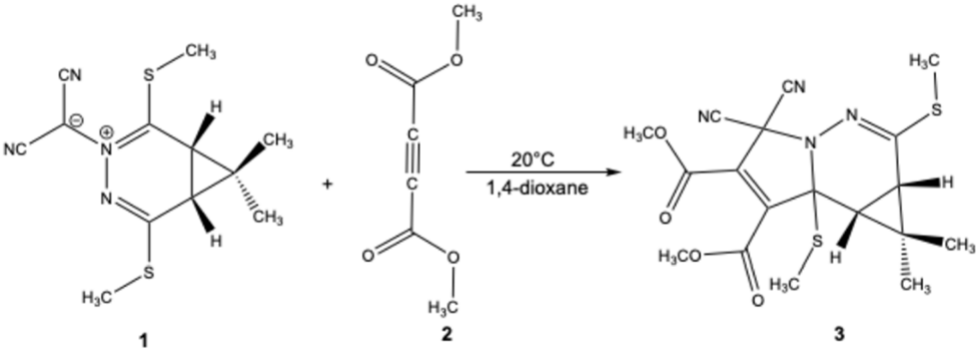
32CA reaction of azomethine ylide 1 with 2pC 2 to yield propargyl *N*-diazabenzpyrrolidine analog 3.

The (3 + 2) cycloaddition (32CA) reaction stands as a pivotal and highly adaptable methodology for the construction of five-membered heterocycles. Its significance stems from its inherent convergent reactivity and extensive applicability across diverse synthetic scenarios.^1–7^ Fundamentally, this reaction centers on the covalent integration of structurally diverse three atom components (TACs) such as azides, nitrones, carbonyl ylides, nitrile oxides, nitrile imines, and azomethine ylides into unsaturated substrates bearing double or triple bonds. Such 32CAs represent a powerful synthetic route for constructing intricate heterocyclic compounds and exploring reactivity pathways.^8–15^ Azomethine ylides, which possess four delocalized electrons over the C–N–C framework, represent a particularly significant class of TAC. Among various dipoles, azomethine ylides have garnered notable attention due to their synthetic utility, prompting ongoing efforts to develop novel ylide precursors and 2-pi-electron components (2pC).^6,16,17^ These ylides are broadly categorized as either non-stabilized (R^1^–R^5^ = H or alkyl) or stabilized forms, with stabilization achieved *via* electron-donating or electron-withdrawing substituents at terminal positions.^17^ Owing to their high reactivity and transient nature, azomethine ylides are typically generated *in situ*, although select stabilized derivatives have been successfully isolated and characterized.^18–20^ The most extensively studied (3 + 2) cycloadditions involving azomethine ylides are those with electron-deficient alkenyl or alkynyl 2-pi-electron component (2pC), affording pyrrolidine derivatives with potential biological activity.^21–25^ The 32CA reactions of stable azomethine ylides with a variety of 2-pi-electron component (2pC) have been extensively studied. Notably, a detailed experimental and kinetic investigation demonstrated that these reactions generally proceed *via* concerted mechanisms, primarily governed by LUMO(ylide)–HOMO(2pC) interactions, with minimal solvent influence on the reaction rate.^26^ These findings support the polar and synchronous nature of such cycloadditions and serve as an important experimental benchmark. More recently, theoretical studies based on density functional theory (DFT) and Molecular Electron Density Theory (MEDT) have further clarified the electronic and mechanistic aspects of these reactions.^27^ These investigations revealed that cyclic azomethine ylides exhibit ambiphilic character and react through asynchronous, one-step polar mechanisms, with activation enthalpies in good agreement with experimental results. Building upon these experimental and theoretical insights, we examined the 32CA reaction between azomethine ylide 1 and dimethyl acetylenedicarboxylate 2, which leads to the formation of the propargyl *N*-diazabenzpyrrolidine analog 3, as depicted in Scheme 1.

In 2016, Domingo introduced Molecular Electron Density Theory (MEDT)^28^ as a conceptual framework to elucidate molecular reactivity based on electron density changes during chemical transformations. Over the past seven years, MEDT has been successfully applied to investigate a wide range of mechanistic features associated with 32CA reactions, including regioselectivity,^29,30^ stereoselectivity,^29–32^ chemoselectivity,^33,34^ global reactivity trends,^35–37^ substituent effects,^38,39^ catalytic behavior,^40,41^ and strain-promoted processes,^42,43^ among others.^44,45^ Central to MEDT is the principle that the redistribution of electron density during a reaction governs the observed reactivity patterns. Within this framework, 32CA reactions are classified into four distinct mechanistic types: (*pdr*) *pseudodiradical*,^46^, (*pmr*) *pseudo(mono)radical*,^47^, (*cb*) *carbenoid*,^48^ and (*zw*) *zwitterionic*,^29^ hence forth referred to as *pdr*, *pmr*, *cb*, and *zw*-type reactions, respectively. These classifications are grounded in the analysis of TACs *via* the topological characterization of monosynaptic electron basins: a basin containing one electron designates a *pseudoradical* center, whereas one containing two electrons indicates a carbenoid center. TACs bearing two *pseudoradical* centers are categorized as *pseudodiradical*, those with a single center as *pseudo(mono)radical*, and those lacking either are considered zwitterionic. This typology allows for establishing a relative reactivity trend of *pdr* > *pmr* > *cb* > *zw*. While *pdr*-type reactions typically proceed rapidly *via* early transition states (TSs), *zw*-type mechanisms generally require additional activation either nucleophilic or electrophilic to facilitate efficient reaction progress. In addition to this TAC-based classification, it is important to emphasize that 32CA reactions may proceed either through a concerted one-step mechanism or *via* a stepwise pathway involving zwitterionic or biradical intermediates.^49^ MEDT provides a comprehensive theoretical framework for analyzing chemical reactivity by integrating various quantum chemical methodologies. Central to this approach is the ELF, initially proposed by Becke and Edgecombe in 1990,^50,51^ stands as a crucial topological tool for probing electron distribution dynamics during chemical transformations. The ELF, *η*(*r*), quantifies the probability of finding an electron in the vicinity of a reference electron with identical spin at a given position *r*. It is rigorously defined by the following expression:1
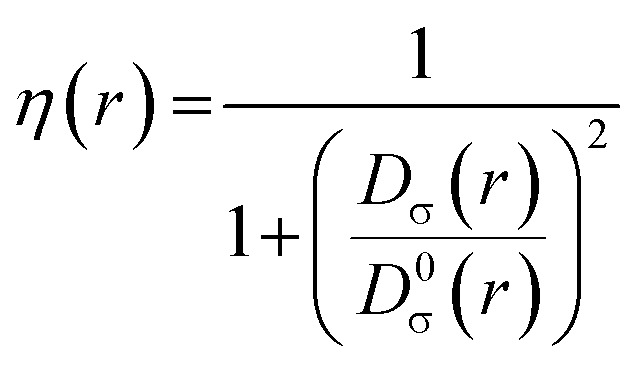
Here, *D*_σ_(r) and *D*_σ_^0^(*r*) are measures of electron localization for the interacting and non-interacting (homogeneous electron gas) systems, respectively. These quantities are given by:

The *D*_σ_(r) and *D*_σ_^0^(*r*) quantities are measures of the electronic localization for ordinary and homogeneous gases, respectively.2
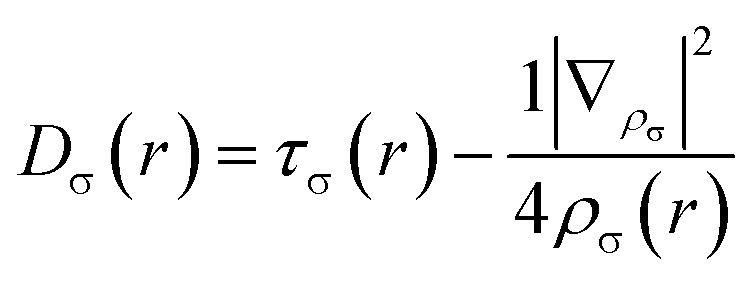
3
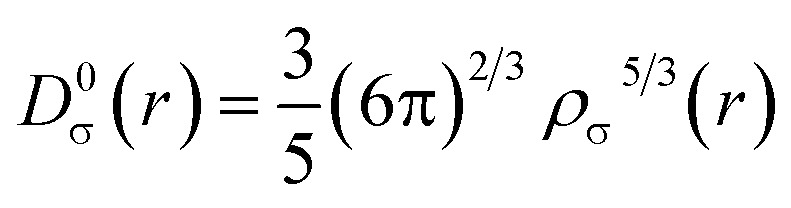
where *ρ*_σ_(*r*) is defined as the electron density for a given *σ* spin |∇_*ρ*_σ__|^2^ and is the kinetic energy.

ELF analysis, meticulously performed along the intrinsic reaction coordinate (IRC), yields Bond Evolution Theory (BET) representations. These representations delineate a chemical reaction as a sequence of discrete chemical events, such as bond formation or cleavage, separated by topological catastrophes. Within this framework, ELF basins, which are regions of maximal probability for finding localized electron pairs, are categorized into core and valence domains. Valence basins are further classified as monosynaptic, disynaptic, or trisynaptic, depending on their association with one, two, or more atomic centers, respectively. Notably, the ELF topological analysis of molecular structures permits the identification of protonated basins (V(A,H)), non-bonding monosynaptic basins (V(A)), and bonding disynaptic basins (V(A,B)), thus providing a comprehensive characterization of electron localization throughout the reaction pathway.^52,53^ Conceptual Density Functional Theory (CDFT) was utilized to clarify and explain the electrical structure and reactivity of the examined systems.^54,55^ The global electrophilicity index (*ω*), an essential parameter in CDFT, is expressed as *ω* = *μ*^2^/2*η*,^56^ where *μ* signifies the electronic chemical potential and *η* indicates the chemical hardness.^55,57^ The parameters were estimated using the energies of the frontier molecular orbitals, with *μ* calculated as (*ε*_H_ + *ε*_L_)/2 and *η* as (*ε*_L_ − *ε*_H_),^58^ where *ε*_L_ and *ε*_H_ represent the lowest unoccupied molecular orbital (LUMO) and the energies of the highest occupied molecular orbital (HOMO), respectively. The nucleophilicity index (*N*) was determined based on the HOMO energies obtained by using the Kohn–Sham formalism to the HOMO energies,^54,59^ it is defined as *N* = *E*_HOMO_(Nu) − *E*_HOMO_(TCE), with tetracyanoethylene (TCE) is used as a reference due to its characteristically low HOMO energy among polar organic molecules.^54^ To further investigate the reactivity profiles of the examined compounds, Mulliken atomic spin density (ASD) calculations were conducted for both radical anion and radical cation species. The computation of the nucleophilic (*P*_k_^−^) and electrophilic (*P*_k_^+^) Parr functions were made easier by these investigations.^60,61^ Afterwards, the local nucleophilicity (*N*_k_) and electrophilicity (*ω*_k_) indices were determined using the formulas *N*_k_ = *N* × *P*_k_^−^ and *ω*_k_ = *ω* × *P*_k_^+^, respectively.^62^ These descriptors enabled a detailed, site-specific evaluation of chemical reactivity within the molecular frameworks.^63^

Bonding Evolution Theory (BET), originally conceptualized by Silvi and Krokidis,^64^ provides a powerful theoretical framework that blends Thom's Catastrophe Theory with quantum chemical principles to elucidate the dynamic evolution of electron density during chemical reactions particularly in regions involving lone-pair-bearing atoms. By tracking the progression of electron localization, BET offers mechanistic insights into bond formation and cleavage events that are often difficult to resolve using conventional approaches.^65–72^ Given that electron density distribution is intimately tied to molecular structure, this evolution is effectively analyzed within the broader context of MEDT.^73–78^ Within BET, chemical transformations are described as sequences of Structural Stability Domains (SSDs), which are topologically invariant regions along the intrinsic reaction coordinate characterized by a fixed number and type of atomic basins. Transitions between SSDs marked by the appearance or disappearance of atomic basins capture the asynchronous, multistage character of processes such as the 32CA reaction. BET fundamentally differentiates between two types of atomic basins: disynaptic basins, V(A,B), which indicate covalent interactions characterized by shared electron density between atoms A and B, and monosynaptic basins, which correspond to regions of localized lone-pair electron density associated with a single atom. Early applications of this approach to the 32CA reaction between 1 and 2 have focused on the formation of key disynaptic interactions, notably the C3–C4 and C5–O1 single bonds. Overall, BET presents a quantifiable and topologically detailed perspective on the electronic structure rearrangements that underpin chemical reactivity, making it a valuable complement to conventional electronic structure analyses. The NCI analysis was conducted through single-point energy calculations on the optimized geometries of the TSs. Subsequently, plots illustrating the relationship between the electron density [*ρ*(*r*)] and the reduced density gradient (RDG) were generated to visualize and characterize the NCI present within the TSs. *s*(*r*) = |∇_*ρ*_(*r*)/2(3π^2^)^1/3^*ρ*(*r*)^4/3^| were obtained by using the NCI Plot program.^79,80^ The Quantum Theory of Atoms in Molecules (QTAIM)^81,82^ was applied to provide comprehensive insight into chemical reactivity. According to the QTAIM, insights into the nature of chemical bonding can be obtained through the analysis of electron density, *ρ*(*r*), and its Laplacian, ∇^2^*ρ*(*r*), at bond critical points (BCPs). A value of *ρ*(*r*) exceeding 0.20 e bohr^−3^ typically indicates a shared-shell interaction, characteristic of covalent bonds, whereas values below 0.10 e bohr^−3^ suggest closed-shell interactions, such as hydrogen bonds or van der Waals forces. In terms of the Laplacian, a negative ∇^2^*ρ*(*r*) reflects electron density accumulation, supporting a covalent nature, while a positive value corresponds to electron density depletion, consistent with noncovalent interactions. Moreover, eqn (4) and (5) describe the calculation of the local kinetic energy density, *G*(*r*), and the potential energy density, *v*(*r*), derived from the electron density, *ρ*(*r*), and its Laplacian, ∇^2^*ρ*(*r*). The combination of these two components, as outlined in eqn (6), yields the total energy density, which serves as a key descriptor in evaluating the electronic environment and bonding characteristics within the QTAIM formalism.4
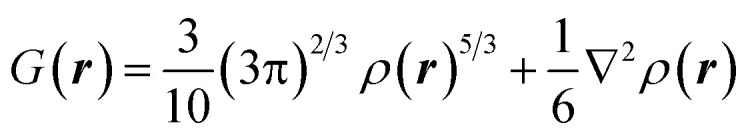
5
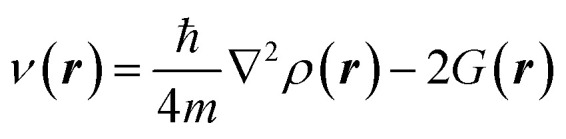

6 ***H***(***r***) = ***G***(***r***) + ***v***(***r***)

The ratio [−***G***(***r***)/***v***(***r***)] is another topological tool to specify the nature of interaction. −***G***(***r***)/*v*(***r***) greater than 1 indicates an interaction stabilized by a local concentration of the charge. Finally, the positive ***H***(***r***) illustrates a shared-shell interaction (covalent bond).

## 2. Computational methods

The electronic structures and fully optimized ground-state geometries of all reactants, products, and TSs were calculated using the B3LYP-D3 exchange–correlation functional combined with the 6-311++G(d,p) basis set. This method offers reliable accuracy in describing structural and electronic features and has been extensively validated in the mechanistic investigation of 32CA reactions, demonstrating consistent performance across recent studies.^83–86^ Vibrational frequency analyses confirmed that the reactants and products reside at true minima on the potential energy surface, as indicated by the lack of imaginary frequencies. In contrast, each TS displayed a single imaginary frequency, confirming its characterization as a first-order saddle point. Thermochemical parameters were then derived from statistical thermodynamic analysis, allowing for the determination of activation and reaction enthalpies, entropies, and Gibbs free energies. All thermodynamic properties were computed under standard conditions (*T* = 293.15 K, *P* = 1 atm). To incorporate solvation effects, the Integral Equation Formalism Polarizable Continum Model (IEFPCM) was applied, using 1,4-dioxane (*ε* = 2.2) as the solvent.^87^ To assess the extent of electronic redistribution in the reaction, we evaluated the Global Electron Density Transfer (GEDT) at the TSs by summing the natural atomic charges (*q*_*i*_) obtained from Natural Bond Orbital (NBO) analysis. The GEDT serves as a robust indicator of charge transfer between molecular fragments, thereby characterizing the polarity and electronic demands inherent to the TS configurations,^88,89^ for all atoms within each defined molecular fragment (f). This is mathematically represented as 
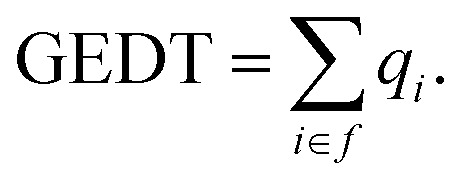
 A positive GEDT value indicates net electron density transfer from the specified framework toward the complementary reacting species. The Intrinsic Reaction Coordinate (IRC) approach was employed to ensure proper connectivity between TSs and corresponding minima,^90^ employing the second-order Gonzalez-Schlegel integration technique,^91,92^ was utilized to meticulously confirm each TS and trace the reaction pathways. The ELF topological analysis was conducted using the TopMod package,^93^ with ELF attractor basin placements visualized through Gauss View 06. To characterize the bonding properties of newly forming bonds in various TSs, QTAIM analysis was performed using the Multiwfn program.^94^ BET-based topological analyses were performed at the B3LYP-D3/6-311++G(d,p) level of theory. At each IRC point, wavefunctions were obtained and subjected to ELF analysis using TopMod with a grid step of 0.2 bohr. Visualization of ELF basins was carried out with DrawMol,^95^ while the progression of basin populations along the IRC was analyzed using DrawProfile.^96^ All calculations were executed using the Gaussian 16 Revision-B.01 software package.^97^

## 3. Results and discussion

The present MEDT-based study is organized into six main sections to provide a comprehensive mechanistic understanding of the 32CA reactions. (i) Initially, ELF topological analysis is employed to examine the ground-state (GS) electronic structures of the reagents 1 and 2. (ii) Subsequently, CDFT descriptors are analyzed to evaluate the polar character and predict the directionality of electronic flux. (iii) The potential energy surface (PES) is then explored along the plausible reaction pathways to identify all stationary points, and to compute the relative enthalpies, entropies, and Gibbs free energies of the TSs and products. In this context, the GEDT values at the TSs are calculated to quantify the polar nature of the reactions. (iv) The electronic structure of the located TSs is further investigated through ELF analysis. (v) The reaction mechanisms of the 32CA reactions involving 1 with 2 are elucidated using the BET framework, offering detailed insight into the sequential changes in bonding along the IRC. (vi) Finally, the electronic structure of the located TSs is further investigated through ELF analysis, while QTAIM and NCI analyses are performed to characterize interatomic interactions.

### 3.1 Investigation of electron localization function topology for 1 and 2

**Fig. 1:**
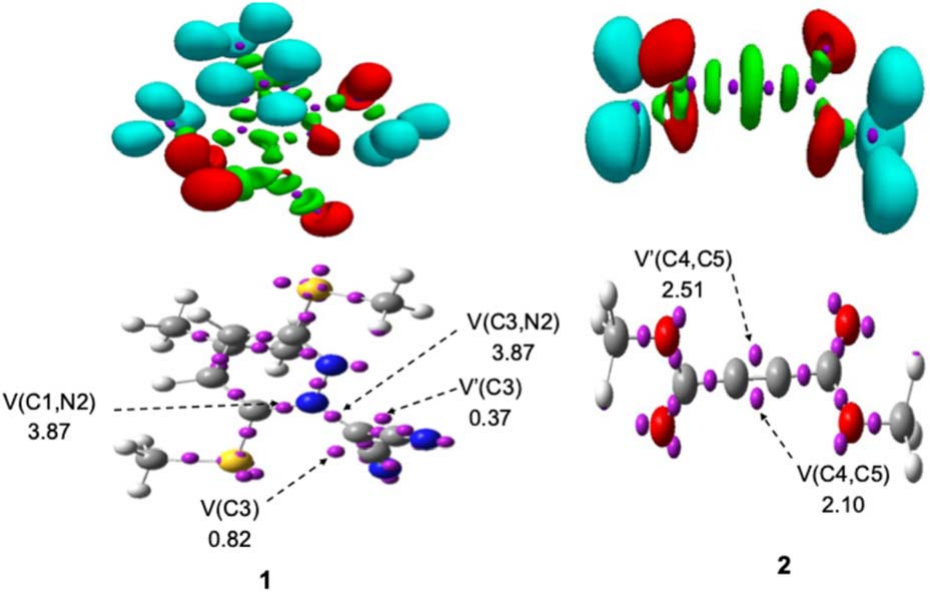
IEFPCM (1,4-dioxane)/B3LYP-D3/6-311++G(d,p) ELF basin attractor positions (isosurface, *η* = 0.75) and the most significant ELF valence basin populations of the reactants 1 and 2.

The ELF analysis serves as a powerful and widely adopted tool for exploring the electronic architecture of TACs in (3 + 2) cycloaddition reactions, offering predictive insights into reactivity by correlating topological features with electronic properties.^98,99^ In this study, a comprehensive ELF topological analysis was performed on the isolated reactants to characterize their valence electron distribution and identify the electronic features that govern their reactivity. Fig. 1 displays the attractor positions and electron populations of the most relevant valence basins identified through ELF analysis. For TAC 1, the ELF topology reveals two monosynaptic basins, V(C3) and V′(C3), localized on the C3 atom, with a combined electron population of 1.19e. Additionally, two disynaptic basins, V(C1,N2) and V(C3,N2), were identified, accumulating a total of 3.87e. These features are consistent with a carbenoid electronic configuration at the C3 center, accompanied by a C1–N2 double bond and a C3–N2 single bond. The presence of less than 2.0e in the monosynaptic basins associated with C3 strongly supports the classification of TAC 1 as a carbenoid-type TAC, suggesting its participation in a carbenoid-type (*cb*-type) 32CA mechanism. In contrast, ELF analysis of 2pC 2 reveals two disynaptic basins, V(C4,C5) and V′(C4,C5), with a combined electron population of 4.61e, indicative of a localized C4≡C5 triple bond. This result highlights the π-rich nature of the 2pC, consistent with its expected reactivity in cycloaddition processes (see Fig. 1).

**Scheme 2:**
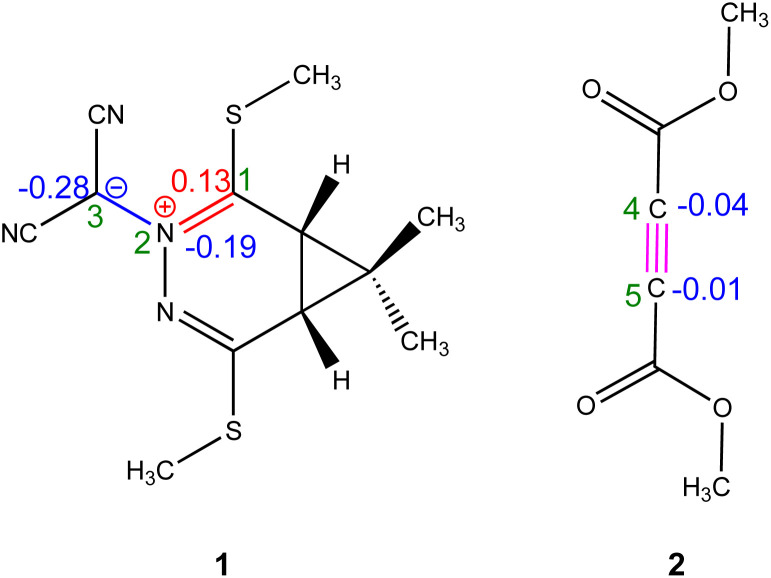
Lewis-like structures of reactants 1 and 2 are depicted with their associated natural atomic charges, represented as average electron e, (in blue color).

Natural Population Analysis (NPA) was performed to investigate the atomic charge distribution of species 1 and 2, as illustrated in Scheme 2.

For 1, the NPA results reveal a marked polarization within the molecular structure: the nitrogen atom (N2) and carbon atom (C3) exhibit significant negative charges of −0.19e and −0.28e, respectively, while the C1 carbon bears a positive charge of +0.13e. This evident charge separation reflects the pronounced electronic asymmetry of 1, consistent with its designation as a carbenoid-type TAC. In contrast, the charge distribution in 2 is comparatively uniform, with C4 and C5 carrying slightly negative charges of-0.04e and −0.01e, respectively. This near-neutral distribution suggests a low degree of polarization across the C≡C bond, affirming the presence of a localized, nonpolar triple bond. Collectively, these findings provide important insight into the electronic factors influencing the reactivity and regioselectivity of the 32CA reaction.

### 3.2 Examination of the CDFT indices for 1 and 2

The CDFT descriptors are key parameters to evaluate the chemical reactivity of a compound participating in a pericyclic reaction.^100–105^ The electronic chemical potential of 1 (−4.08 eV) is higher than that of 2 (−5.47 eV) (Table 1), indicating that the GEDT in this 32CA reaction will take place from 1 to 2. In addition, according to the electrophilicity and nucleophilicity scales,^106^ 1 is a strong electrophile (*ω* = 2.68 eV) and a strong nucleophile (*N* = 3.84 eV). While 2 (*ω* = 2.34 eV, *N* = 1.08 eV) being classified as a strong electrophile and a low nucleophile (Table 1). The super-nucleophilic character of 1, together with the strong electrophilic character of 2, indicates that the corresponding 32CA reaction will have a high polar character, being classified as of forward electron density flux (FEDF).^107^

**Table 1:** IEFPCM(1,4-dioxane)/B3LYP-D3/6–311++G(d,p) electronic chemical potential (*μ*), chemical hardness (*η*), electrophilicity index (*ω*), and nucleophilicity index (*N*) for reactants 1 and 2

Reactants	*ε*_H_	*ε*_L_	*μ*	*η*	*ω*	*N*
1	−5.63	−2.53	−4.08	3.10	2.68	3.84
2	−8.39	−2.00	−5.47	6.39	2.34	1.08

**Fig. 2:**
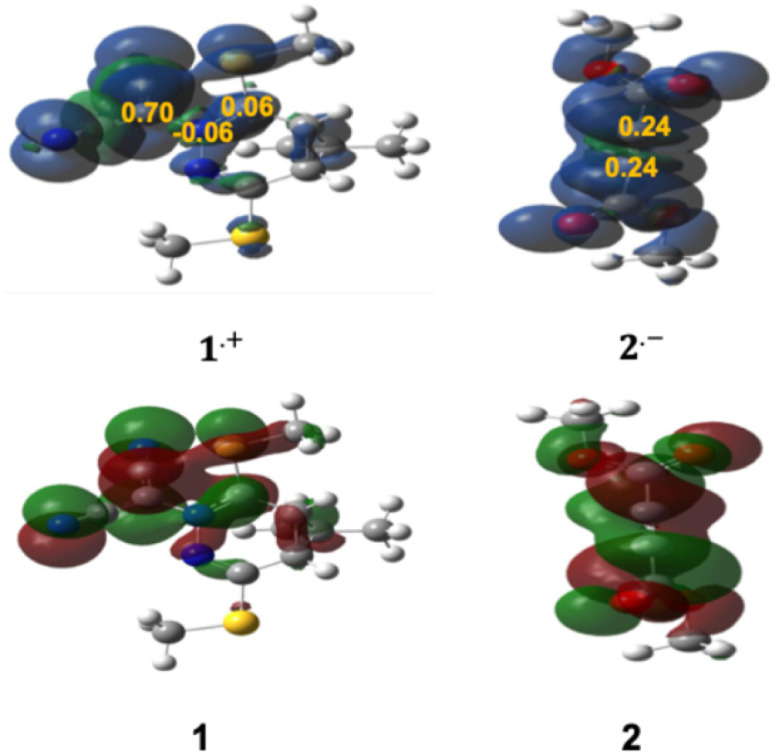
3D visualizations of the ASD at an isovalue of (*η* = 0.02) were obtained for the radical cation 1˙^+^ and the radical anion 2˙^−^ using the IEFPCM(1,4-dioxane)/B3LYP-D3/6–311++G(d,p) computational level. These representations are analyzed alongside the nucleophilic Parr functions (*P*_k_^−^) of 1 and the electrophilic Parr functions (*P*_k_^+^) of 2, in comparison to the HOMO of 1 and the LUMO of 2.

Local reactivity indices, including spin and charge density-based Parr functions (*P*_k_^−^ for nucleophilic and *P*_k_^+^ for electrophilic sites), offer detailed insights into specific reactive centers within molecules involved in cycloaddition reactions. These descriptors significantly enhance the predictive capabilities of CDFT, allowing for improved rationalization and alignment with experimental observations in organic synthesis. In this context, analysis of *P*_k_^−^ and *P*_k_^+^ functions derived from the spin electron densities of radical cations and anions through the GEDT, rank among the most reliable descriptors for assessing local reactivity in polar and ionic processes. Accordingly, based on the nature of the reactants, a detailed analysis of the nucleophilic *P*_k_^−^ Parr functions of 1 and the electrophilic *P*_k_^+^ Parr functions of 2 was conducted (see Fig. 2 and Table S1). The C1 and C3 carbon atoms of 1 exhibit nucleophilic activation with *P*_k_^−^ values of 0.06 and 0.70, respectively, identifying C3 as the most nucleophilic center within 1. Conversely, the N2 nitrogen atom shows nucleophilic deactivation with a *P*_k_^−^ value of −0.06. For 2, examination of the electrophilic *P*_k_^+^ Parr functions reveals that the C4 and C5 carbon atoms are the most electrophilic centers, both having a *P*_k_^+^ value of 0.24. Consequently, it is anticipated that the most favorable nucleophilic/electrophilic interaction during this 32CA reaction will occur between the C3 atom of 1 and the C4 atom of 2. This suggests that the initial interaction between the HOMO of 1 and the LUMO of 2 will lead to the formation of the C3–C4 bond, followed by the formation of the C1–C5 bond. This conclusion is further supported by findings presented in the BET section.

### 3.3 Analysis of the energy summary associated with the 32CA reactions of 1 and 2

**Scheme 3:**
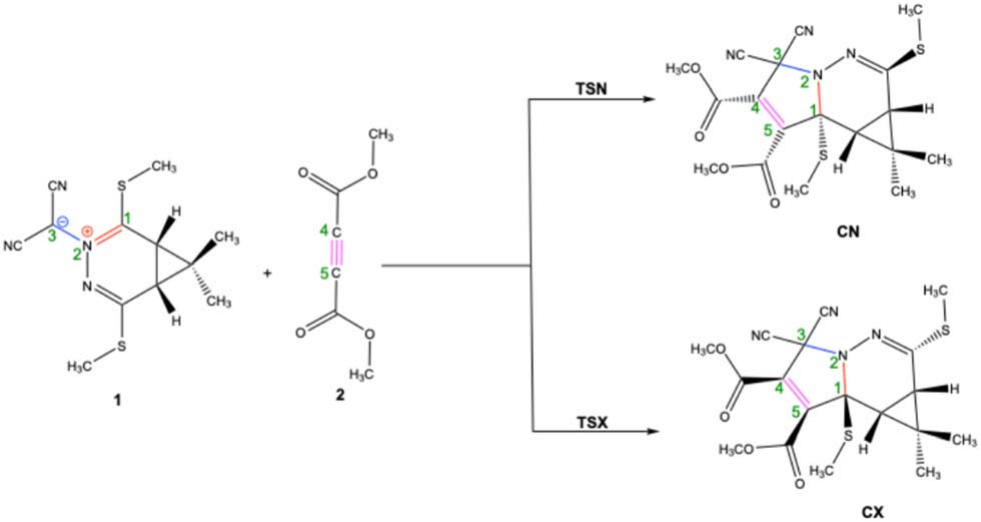
32CA reactions stereoisomeric paths of azomethine ylide 1 with dimethyl acetylenedicarboxylate 2. [CN = *endo* cycloadduct, CX = *exo* cycloadduct, TSN = TS-*endo*, and TSX = TS-*exo*].

The 32CA reaction between 1 and 2 proceeds *via* two distinct stereoisomeric pathways: *endo* and *exo*. Computational analysis of the potential energy surface identified corresponding TSs, denoted as TS-*endo* (TSN) and TS-*exo* (TSX), which lead to the formation of two cycloadducts, CN (*endo* cycloadduct) and CX (*exo* cycloadduct), respectively. The presence of a single TS for each stereoisomeric pathway strongly indicates a one-step, concerted mechanism for this 32CA reaction, as depicted in Scheme 3. The calculated enthalpies (Δ*H*), entropies (Δ*S*), and Gibbs free energies (Δ*G*), along with their relative values, are presented in Table 2.

**Table 2:** Thermochemical parameters of the stationary points involved in the 32CA reaction of 1 and 2 Calculated at the IEFPCM(1,4-dioxane)/B3LYP-D3/6-311++G(d,p) level of theory, as well as the relative electronic energies (Δ*E*, in kcal mol^−1^), relative enthalpies (Δ*H*, in kcal mol^−1^), Gibbs free energies (Δ*G*, in kcal mol^−1^), and entropies (Δ*S*, in cal mol^−1^ K^−1^)

Reactions	Δ*E*	Δ*H*	Δ*S*	Δ*G*
CX	−47.00	−44.90	−57.35	−28.10
CN	−51.34	−49.15	−53.08	−33.59
TSX	7.05	7.24	−50.95	22.18
TSN	9.66	9.57	−50.30	24.34

**Fig. 3:**
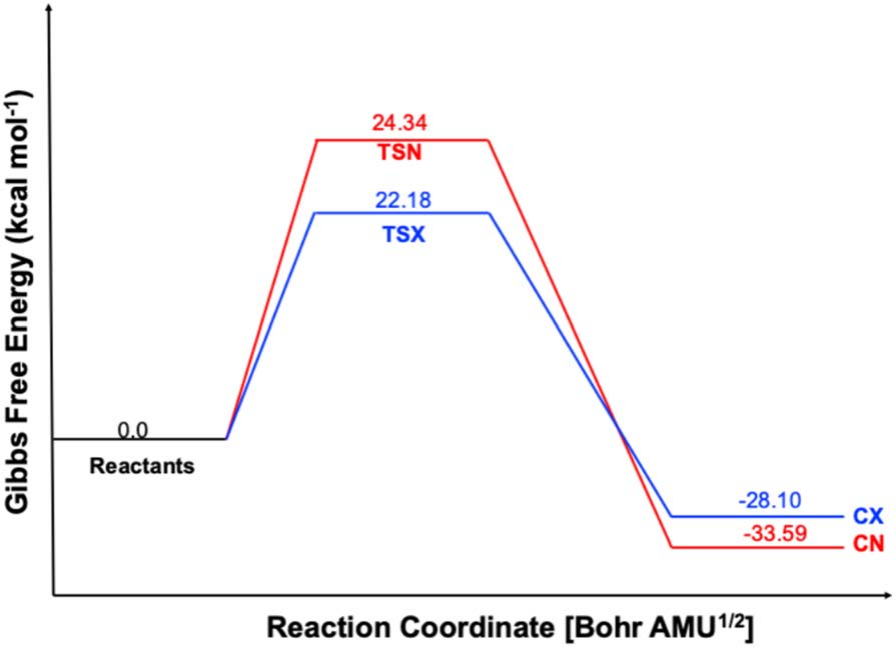
Gibbs free energy profiles (Δ*G*, expressed in kcal mol^−1^) for each stationary point along the path CN and CX, were assessed with various IEFPCM(1,4-dioxane)/B3LYP-D3/6-311++G(d, p).

Table 2 clearly demonstrates that the reaction is both highly exothermic and exergonic, as indicated by the negative Δ*H* and Δ*G* values for the formation of both CN and CX products (Fig. 3). Notably, the CN product is thermodynamically preferred, exhibiting significantly lower electronic energy (Δ*E* = −51.34 kcal mol^−1^), enthalpy (Δ*H* = −49.15 kcal mol^−1^), and Gibbs free energy (Δ*G* = −33.59 kcal mol^−1^) compared to the CX product (−47.00, −44.90, and −28.10 kcal mol^−1^, respectively). This enhanced thermodynamic stability of CN is reflected in the equilibrium distribution at 293.15 K, where it constitutes approximately 97.9% of the product mixture, compared to only 2.1% for CX. In contrast, the CX pathway is kinetically favored due to a lower activation Gibbs free energy (Δ*G* = 22.18 kcal mol^−1^), which is about 2.2 kcal mol^−1^ less than that of the CN pathway (Δ*G* = 24.34 kcal mol^−1^). This difference translates into an approximately tenfold faster formation rate for the CX product. Consequently, the reaction displays a classic case of kinetic *versus* thermodynamic control, where the *exo* (CX) product forms more rapidly under kinetic conditions, but the *endo* (CN) product, being thermodynamically more stable, predominates at equilibrium. This interplay explains the observed strong *endo*-selectivity in the final product distribution.

The optimized geometries of TSs, highlighting the forming C1–C5 and C3–C4 bonds, to evaluate the extent of asynchronicity in the investigated 32CA reaction pathways, a detailed diagnostic analysis was performed on all key stationary points.^108^ Specifically, key parameters including the interatomic distances between reactive centers (*r*), the bond development index (*l*), and the bond formation asynchronicity index (Δ*l*) were calculated and are compiled in Table 3. These descriptors offer valuable insight into the synchronicity of bond formation for both the *endo* and *exo* reaction pathways.

**Table 3:** IEFPCM(1,4-dioxane)/B3LYP-D3/6-311++G(d,p) key parameters of critical structures for the studied 32CA reaction^a^

	*r*_C1–C5_ (Å)	*l*_C1–C5_	*r*_C3–C4_ (Å)	*l*_C3–C4_	Δ*l*
TSN	2.376	0.428	2.321	0.498	0.07
CN	1.511		1.545		
TSX	2.505	0.348	2.100	0.635	0.287
CX	1.516		1.538		

a
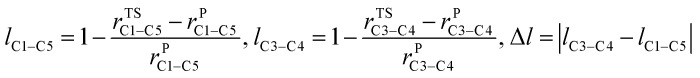

**Fig. 4:**
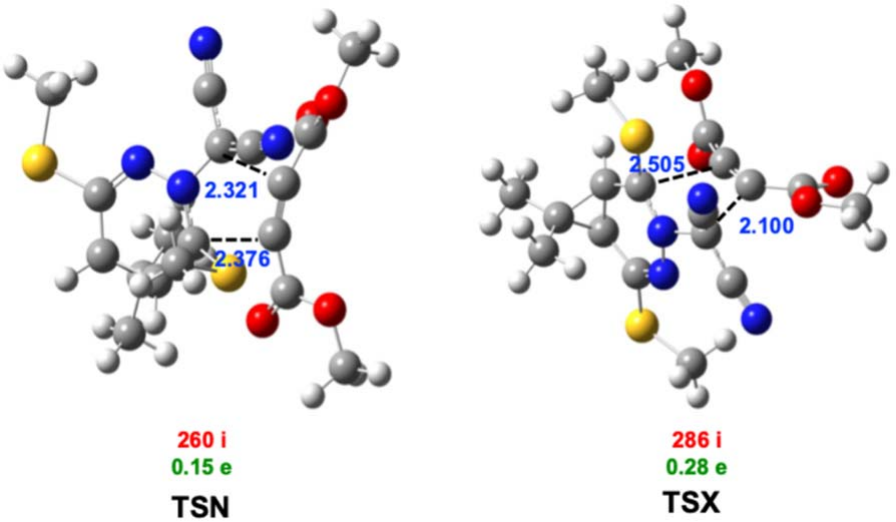
IEFPCM(1,4-dioxane)/B3LYP-D3/6-311++G(d,p) optimized geometries of the TSs involved in the 32CA reaction of 1 and 2. The C1–C5 and C3–C4 distances values are given in Å, are provided in blue. The characteristic imaginary frequencies associated with TSs, expressed in cm^−1^, are shown in red. The GEDT values at TSs, calculated as the electronic charge transferred to 2 in e, are presented in green.

The TSs associated with both *endo* (TSN) and *exo* (TSX) pathways exhibit C1–C5 and C3–C4 bond lengths that exceed those in the final cycloadducts yet remain within the typical range for 32CA transition structures. Analysis of bond development indices (*I*) reveals values of 0.428 (TSN) and 0.348 (TSX) for the nascent C3–C4 bonds, and 0.498 (TSN) and 0.635 (TSX) for the C1–C5 bonds. These results suggest that C3–C4 bond formation precedes that of C1–C5 in both TSs. The corresponding asynchronicity indices (Δ*l*) are 0.07 for TSN and 0.287 for TSX, confirming an asynchronous bond formation pattern in both cases, with a more pronounced asynchronicity observed for the *exo* pathway. To further characterize the reaction mechanism, the GEDT values at the TSs were evaluated (Fig. 4). According to Domingo's criteria, GEDT values approaching 0.0e indicate nonpolar reaction, while values exceeding 0.2e suggest polar processes.^109^ The calculated GEDT values of 0.15e (TSN) and 0.28e (TSX) imply a moderate polar character for the *endo* (TSN) and a distinctly polar character for the *exo* (TSX). The electron density flux proceeds from the TAC 1 to 2pC 2, in agreement with the higher chemical potential of 1 (−4.08 eV) compared to 2 (−5.47 eV) (Table 1). Consequently, this reaction is classified as FEDF reactions.^107^

### 3.4 Topological analysis of the TSs along the 32CA reactions when O1–C5 or C3–C4

**Fig. 5:**
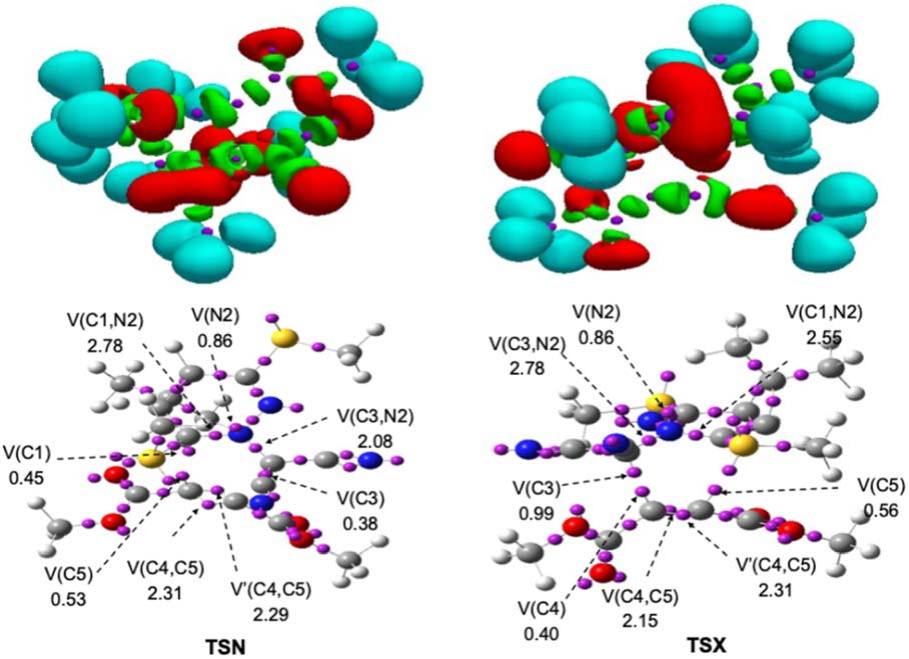
Basin attracter positions for the TSs associated with 32CA reaction between 1 and 2, computed at the B3LYP-D3/6-311G++ (d,p) level of theory. The ELF domain (isosurface value = 0.80), protonated basins are depicted in blue, monosynaptic basins in red, disynaptic basins in green, and core basins in purple.

The TSN and TSX, were rigorously characterized through a topological analysis of the ELF. As depicted in Fig. 5, protonated basins are represented in blue, disynaptic basins (signifying shared-electron interactions) in green, and monosynaptic basins (indicating lone pairs or radical centers) in red, with color breaks denoting attractor positions. The ELF analysis consistently revealed a V(N2,C3) disynaptic basin, corresponding to the N2–C3 bonding region, which integrated electron populations of 2.78e in TSN and 2.55e in TSX. Similarly, the V(N2,C1) disynaptic basin was present in both TSN and TSX, each exhibiting a total electron population of 2.78e, underscoring a consistent bonding interaction in this region. Both TSs also featured a V(N2) monosynaptic basin, associated with the non-bonding electron density at the nitrogen atom, each integrating approximately 0.86e. Furthermore, the V(C4,C5) disynaptic basin, indicative of a robust bonding interaction between the C4 and C5 atoms, showed integrated electron populations of 4.60e in TSN and 4.46e in TSX. Monosynaptic basins were also identified at C5, integrating 0.53e in TSN and 0.56e in TSX, and at C4, with populations of 0.45e in TSN and 0.40e in TSX. Notably, TSN exhibited a monosynaptic basin at C1 with a population of 0.45e, while TSX displayed one at C3 with 0.99e. The pervasive presence of these monosynaptic basins on carbon centers, coupled with the partial electron populations observed in the relevant disynaptic regions, strongly indicates that complete covalent bond formation between the C3–C4 and C1–C5 centers has not yet occurred in either TS. This comprehensive ELF analysis thus firmly characterizes both TSN and TSX as early-stage, asynchronous transition structures, providing crucial insights into their electronic nature during the bond-forming processes.

### 3.5 Analysis of the molecular mechanism of the 32CA reaction *via* BET

**Fig. 6:**
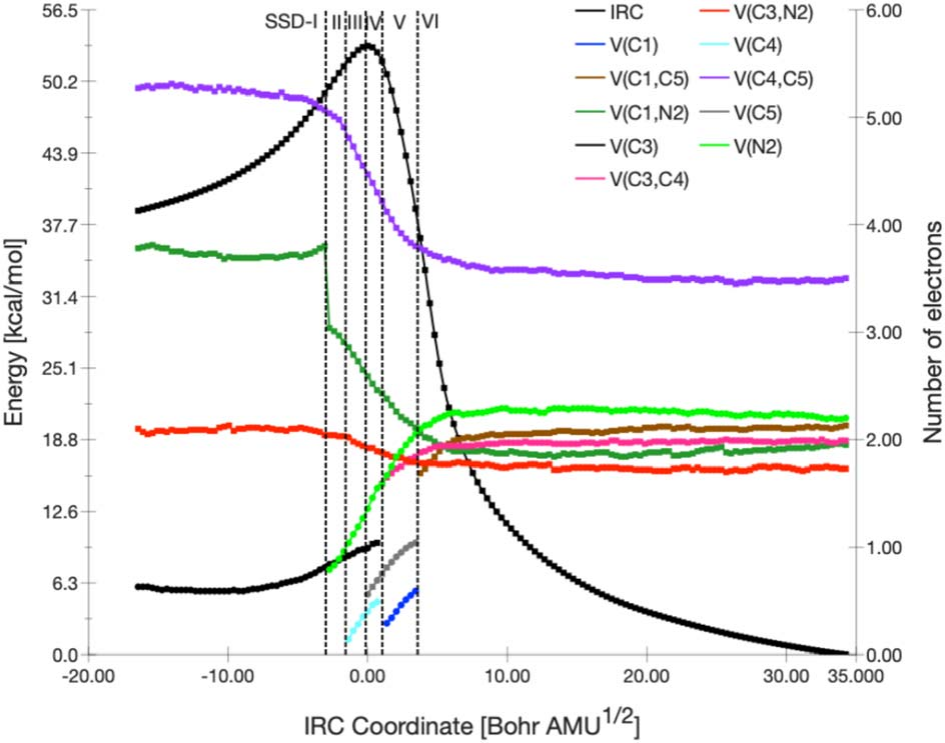
Population evolution (in e) of selected basins along the TSX reaction path of the 32CA reaction between 1 and 2 as evaluated at the IEFPCM(1,4-dioxane)/B3LYP/6-311++G(d,p) level of theory. The relative potential energy surface corresponds to the black dot curve.

**Fig. 7:**
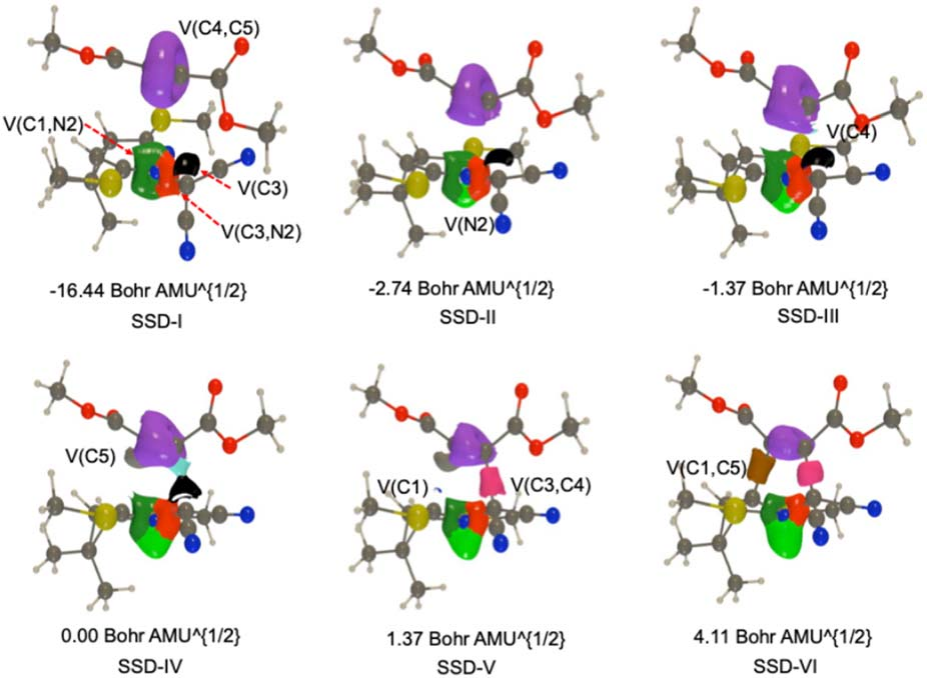
ELF (*η* = 0.72) snapshots of selected basins related to the bond forming process along the TSX reaction path.

In the 32CA reaction between 1 and 2 by the TS*-exo* stereoisomeric pathway, the BET analysis reveals a total of six structural stability domains (SSDs) during the reaction as shown in Fig. 6 and 7, which summarizes the variation in the basin populations that participate in this reaction. The SSD-I [d(C1–C5) = 3.37 Å and d(C3–C4) = 3.47 Å] visualize the ELFs topologies of the reactants reported in the Table S1. 1 display the following basins: one monosynaptic V(C3) basin that is related to the carbenoid center at C3 with a total population of 0.63e and two disynaptic V(C1,N2), and V(C3,N2) basins covering 3.78, and 2.10e, respectively, in agreement with double and single (C1–N2) and (C3–N2) bonds. Meanwhile, the 2 frameworks show one disymmetric basin related to the C4–C5 triple bond V(C4,C5) is observed with an electron population of 5.27e. SSD-I. The reduction in the V(C1,N2) basin population starts at the SSD-II[d(C1–C5) = 2.77 Å and d(C3–C4) = 2.56 Å], to attain 3.04e due to the formation of a new monosynaptic basin, V(N2), with a total population of 0.79e, resulting from a fold-F type catastrophe. In the SSD-III domain [d(C1–C5) = 2.64 Å and d(C3–C4) = 2.36 Å, the population of the V(C4,C5) basin begins to diminish. This reduction triggers an *F* catastrophe, leading to the emergence of a new monosynaptic V(C4) basin, which holds 0.14e. By the end of this domain, the populations of the V(C3), V(N2), and V(C4) basins reach 0.98e, 1.27e, and 0.35e, respectively. Further into SSD-VI (d(C1–C5) = 2.51 Å and d(C3–C4) = 2.15 Å), the V(C4,C5) basin's population continues to decrease due to a fold-F catastrophe, which results in the formation of another new monosynaptic basin, V(C5), with a population of 0.56e. In the SSD-V domain (d(C1–C5) = 2.39 Å and d(C3–C4) = 1.99 Å), the two monosynaptic basins, V(C3) and V(C4), coalesce *via* a cusp-C type catastrophe to form the new disynaptic V(C3,C4) basin, holding 1.59e. Simultaneously, an additional F-type catastrophe arises within this domain, resulting in the formation of a monosynaptic V(C1) basin with an electron population of 0.29e. This basin emerges due to the continuous depletion of the disynaptic V(C1,N2) basin. The final topological transformation occurs in the SSD-VI domain [d(C1–C5) = 1.98 Å and d(C3–C4) = 1.65 Å], where a C-type catastrophe leads to the emergence of the disynaptic V(C1,C5) basin, signifying the formation of the C1–C5 bond. At the onset of SSD-VI, this newly formed basin integrates 1.69e, primarily derived from the disappearance of the monosynaptic V(C1) and V(C5) basins.

### 3.6 NCI and QTAIM topological analysis at TSs

**Fig. 8:**
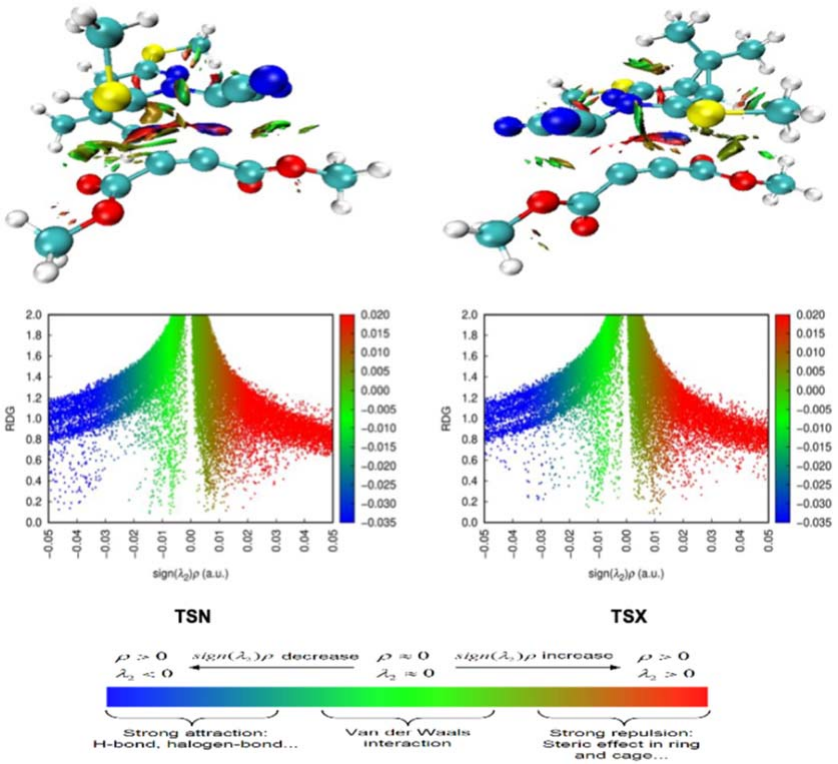
NCI and reduced density gradient mapping of the TSs (TSN, and TSX).

NCI significantly influence the stereoselectivity of reactions such as the 32CA process. Fig. 8 presents scatter plots of the RDG *versus* sign(*λ*_2_)*ρ* for the two TSs, TSX and TSN, providing insight into the nature and intensity of non-covalent interactions in each case. These plots differentiate between attractive interactions (negative *λ*_2_, typically shown in blue or green) and repulsive interactions (positive *λ*_2_, shown in red). In TSX, the RDG plot reveals a dominant blue region between −0.05 and −0.02 a.u., indicative of strong localized attractive interactions, primarily of electrostatic nature. Additionally, a narrow green region corresponds to weak van der Waals forces, while a visible red zone reflects steric repulsion. These features are consistent with the 3D RDG isosurface, which shows an intense blue region between the interacting nitrone carbon atoms, highlighting directional and stabilizing interactions that help lower the activation barrier. Conversely, TSN displays a broader green region and a less intense blue zone, pointing to weaker, more diffuse van der Waals interactions and a reduced degree of electrostatic stabilization. The corresponding RDG isosurface shows more dispersed green zones, reflecting a less compact TS structure with less effective orbital overlap. These differences in non-covalent interaction patterns correlate directly with the calculated activation barriers, where TSX is favored kinetically due to stronger stabilizing interactions that lower its activation Gibbs free energy. This interpretation aligns with the irreversible character of the reaction, as shown in Fig. 3, where the product distribution is dictated by the relative transition state energies. In summary, the stereoselectivity arises under kinetic control, and the NCI analysis confirms that the faster forming *exo* product (CX) results from stronger and more localized attractive interactions in TSX. Despite the weaker interactions in TSN leading the higher barrier, the greater thermodynamic stability of the *endo* product (CN) gives its high formation rate. The product formed *via*TSX is kinetically favored due to the presence of strong localized attractive interactions that lower the activation barrier despite moderate steric repulsion, whereas the product formed *via*TSN, less accessible kinetically, is thermodynamically more stable owing van der Waals interaction stabilization.

**Fig. 9:**
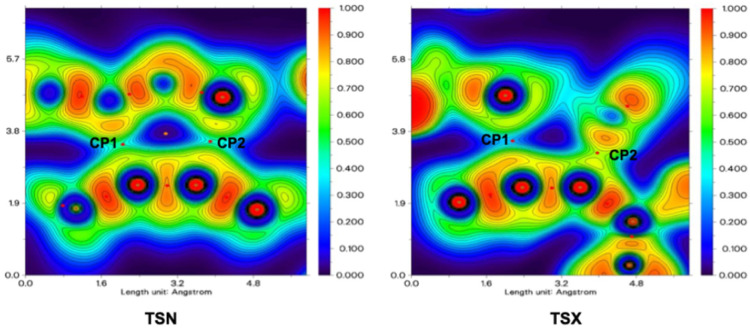
3D drawings of the ELF's color-filled maps in the C1–C4 and C3–C5 molecular planes of TSN, and TSX. Red is used to show (3, −1) cps with ∇^2^*ρ*_cp_ < 0.

Table 4 summarizes the topological parameters derintensity of non-ived from the electron density and its Laplacian, specifically at the C3–C4 and C1–C5 bond critical points, for the TSX and TSN. These values were obtained through calculations performed at the IEFPCM-B3LYP-D3/6-311G++(d,p) level of theory. In TSX, the electron density at the C1–C5 interaction is higher than at C3–C4, suggesting that bond formation is more advanced at the C1–C5 site. The Laplacian values follow a similar trend, with the lower ∇^2^*ρ* value at C1–C5 indicating a more covalent character. In contrast, TSN exhibits higher electron density at C3–C4 than at C1–C5, showing a different bonding progression. The more balanced Laplacian values in TSN imply that both bonding interactions are at comparable stages. These differences reflect varying degrees of asynchronicity in bond formation between the two TSs. Fig. 9 presents the color-mapped representations of both the Laplacian of the electron density and the Electron Localization Function (ELF) plotted within the molecular plane defined by the O1, C5, C3, and C4 nuclei. These visualizations offer valuable insight into the electronic structure and localized bonding interactions within the specified region.

**Table 4:** Presents a comprehensive topological analysis of the forming C3–C4 and C1–C5 bonds within the TSs. It includes the bond development indices, bond distances (in Å), and the values of both the electron density (*ρ*, in atomic units) and its Laplacian (∇^2^*ρ*(*r*)), (in atomic units) at the respective bond critical points

Structure	CP1 (C3–C4)		CP2(C1–C5)	
TS_s_	ρ	∇^2^p (r)	ρ	∇^2^p (r)
TSX	0.03356	0.05084	0.07538	0.02322
TSN	0.04294	0.04519	0.04803	0.04530

## 4. Conclusion

This study provides a detailed mechanistic understanding of the (3 + 2) cycloaddition reaction between azomethine ylide 1 and dimethyl acetylenedicarboxylate 2 through a multi-faceted DFT approach. Our computational protocol, employing the B3LYP-D3 functional with the 6-311G ++(d,p) basis set and IEFPCM solvent (1,4-dioxane) model, reliably characterized the stationary points and reaction pathways. Key findings from this investigation include that CDFT global and local reactivity indices consistently indicated a highly polar reaction with a forward electron density flux (FEDF) from azomethine ylide 1 to dimethyl acetylenedicarboxylate 2, with NPA and Parr function analyses pinpointing the C3 atom of 1 and the C4/C5 atoms of 2 as the most reactive centers for initial bond formation. The reaction is thermodynamically favorable, as indicated by its highly exothermic and exergonic profile. Although kinetic analysis shows a preference for the *exo* pathway due to its lower activation barrier, the more stable *endo* product predominates experimentally, suggesting a reversible process under thermodynamic control. Analysis of TSs geometries and bond development indices confirmed the asynchronous nature of bond formation for both *endo* and *exo* pathways, with the *exo* pathway exhibiting a more pronounced asynchronicity. Furthermore, GEDT values underscored the polar character of the TSs. Mechanistic insights from electron density, gained through ELF topological analysis of the TSs, identified early-stage, asynchronous structures with persistent monosynaptic basins on carbon centers, suggesting incomplete bond formation. The Bonding Evolution Theory (BET) analysis further revealed a complex, six-stage molecular mechanism for the *exo* pathway, characterized by a series of topological catastrophes (fold-F and cusp-C type) that delineate the sequential formation of the C3–C4 and C1–C5 bonds, offering a clear, quantitative picture of bond formation and rupture along the reaction coordinate. Lastly, NCI analysis visually and quantitatively confirmed the presence and nature of non-covalent interactions within the TSs, highlighting their role in stabilizing these crucial intermediates. Overall, this study integrates various computational methodologies to provide a comprehensive and atomic-level understanding of the (3 + 2) cycloaddition reaction, unraveling the intricate electronic and energetic factors that dictate its reactivity, selectivity, and mechanism. The insights gained contribute significantly to the broader understanding of cycloaddition chemistry and can aid in the rational design of new synthetic strategies.

## Author contributions

Mohamed Chellegui, Raghad Mowafak Al-Mokhtar, Raad Nasrullah Salih, Lakhdar Benhamed, and Sofiane Benmetir: writing, investigation, validation, methodology; Haydar A. Mohammad-Salim, Jesus Vicente de Julián-Ortiz: validation, supervision; Ali Ben Ahmed: editing, reviewing, and supervision. All authors have read and approved the published version of the manuscript.

## Conflicts of interest

There are no conflicts to declare.

## Supplementary Material

RA-015-D5RA04992J-s001

## Data Availability

The data supporting this article have been included as part of the SI. Supplementary information is available. See DOI: https://doi.org/10.1039/d5ra04992j.

## References

[cit1] Wong G., Howell M., Patrick E., Yang J. (2017). Taking kidneys for granted? Time to reflect on the choices we make. Transplantation.

[cit2] Hashimoto T., Maruoka K. (2015). Recent advances of catalytic asymmetric 1,3-dipolar cycloadditions. Chem. Rev..

[cit3] Usova S. D., Knyazeva E. A., Rakitin O. A. (2024). Cyclopent-4-ene-1,3-diones fused with heterocycles as promising anchor groups in non-fullerene acceptors (microreview). Chem. Heterocycl. Compd..

[cit4] Wang L. J. and Tang Y., Intermolecular 1,3-Dipolar Cycloadditions of Alkenes, Alkynes, and Allenes, Elsevier Ltd, 2014, 10.1016/B978-0-08-097742-3.00428-6

[cit5] Menon R., Nair V. (2014). Intramolecular 1,3-dipolar cycloadditions of alkenes, alkynes, and allenes. Compr. Org. Synth. (2nd Ed.).

[cit6] Chaban T. I., Lelyukh M. I., Chaban I. H., Kasyanchuk O. Y. (2024). Approaches to the synthesis of thiazolo [3,2-a] pyridines (microreview). Chem. Heterocycl. Compd..

[cit7] Bondarenko S. S., Lega D. A. (2024). Recent advances in the chemistry of pyrrolidine-2,3-diones (microreview). Chem. Heterocycl. Compd..

[cit8] Izquierdo C., Esteban F., Ruano J. L. G., Fraile A., Alemán J. (2016). Asymmetric synthesis of 1,2-diamines bearing tetrasubstituted centers from nonstabilized azomethine ylides and n-sulfinylketimines under brønsted acid catalysis. Org. Lett..

[cit9] Yang H., Seela F. (2016). Circular DNA by “bis-click” ligation: template-independent intramolecular circularization of oligonucleotides with terminal alkynyl groups utilizing bifunctional azides. Chemistry.

[cit10] Cochrane S. A., Li X., He S., Yu M., Wu M., Vederas J. C. (2015). Synthesis of tridecaptin-antibiotic conjugates with *in vivo* activity against gram-negative bacteria. J. Med. Chem..

[cit11] Nakhla M. C., Lee C.-W., Wood J. L. (2015). Chemoselective intramolecular carbonyl ylide formation through electronically differentiated malonate diesters. Org. Lett..

[cit12] Haberhauer G., Gleiter R., Woitschetzki S. (2015). Anti-diradical formation in 1,3-dipolar cycloadditions of nitrile oxides to acetylenes. J. Org. Chem..

[cit13] Hoogenboom J., Zuilhof H., Wennekes T. (2015). Versatile scope of a masked aldehyde nitrone in 1,3-dipolar cycloadditions. Org. Lett..

[cit14] Hatano J., Okuro K., Aida T. (2016). Photoinduced bioorthogonal 1,3-dipolar poly-cycloaddition promoted by oxyanionic substrates for spatiotemporal operation of molecular glues. Angew. Chem., Int. Ed. Eng..

[cit15] Brioche J., Meyer C., Cossy J. (2015). Synthesis of 2-aminoindolizines by 1,3-dipolar cycloaddition of pyridinium ylides with electron-deficient ynamides. Org. Lett..

[cit16] Maas G. (2003). Synthetic applications of 1,3-dipolar cycloaddition chemistry toward heterocycles and natural products. Cheminform.

[cit17] Pandey G., Banerjee P., Gadre S. R. (2006). Construction of enantiopure pyrrolidine ring system *via* asymmetric [3+2]-cycloaddition of azomethine ylides. Chem. Rev..

[cit18] Huisgen R. (1984). Ozonation in Organic Chemistry. Angew. Chem..

[cit19] Song G., Chen D., Su Y., Han K., Pan C.-L., Jia A., Li X. (2011). Isolation of azomethine ylides and their complexes: iridium(iii)-mediated cyclization of nitrone substrates containing alkynes. Angew. Chem., Int. Ed. Eng..

[cit20] Lee D. J., Han H. S., Shin J., Yoo E. J. (2014). Multicomponent [5 + 2] cycloaddition reaction for the synthesis of 1,4-diazepines: isolation and reactivity of azomethine ylides. J. Am. Chem. Soc..

[cit21] Coldham I., Hufton R. (2005). Intramolecular dipolar cycloaddition reactions of azomethine ylides. Chem. Rev..

[cit22] Husinec S., Savic V. (2005). Chiral catalysts in the stereoselective synthesis of pyrrolidine derivatives *via* metallo-azomethine ylides. Tetrahedron:Asymmetry.

[cit23] Nájera C., Sansano J. M. (2005). Catalytic enantioselective 1,3-dipolar cycloaddition reaction of azomethine ylides and alkenes: the direct strategy to prepare enantioenriched highly substituted proline derivatives. Angew. Chem., Int. Ed. Eng..

[cit24] Pearson W., Stoy P. (2003). Cycloadditions of nonstabilized 2-azaallyllithiums (2-azaallyl anions) and azomethine ylides with alkenes: [3 + 2] approaches to pyrrolidines and application to alkaloid total synthesis. Synlett.

[cit25] Koumbis A., Gallos J. (2005). 1,3-Dipolar cycloadditions in the synthesis of carbohydrate mimics. part 3: azides, diazo compounds and other dipoles. Curr. Org. Chem..

[cit26] Elender K., Riebel P., Weber A., Sauer J. (2000). 1,3-Dipolar cycloaddition reactions of stable bicyclic and monocyclic azomethine ylides: kinetic aspects. Tetrahedron.

[cit27] Domingo L. R., Kula K., Ríos-Gutiérrez M. (2020). Unveiling the reactivity of cyclic azomethine ylides in [3+ 2] cycloaddition reactions within the molecular electron density theory. Eur. J. Org Chem..

[cit28] Domingo L. (2016). Molecular electron density theory: a modern view of reactivity in organic chemistry. Molecules.

[cit29] Domingo L. R., Ríos-Gutiérrez M., Pérez P. (2018). A molecular electron density theory study of the reactivity and selectivities in [3 + 2] cycloaddition reactions of C, N-dialkyl nitrones with ethylene derivatives. J. Org. Chem..

[cit30] Domingo L. R., Ríos-Gutiérrez M., Adjieufack A. I., Ndassa I. M., Nouhou C. N., Mbadcam J. K. (2018). Molecular electron density theory study of fused regioselectivity in the intramolecular [3+2] cycloaddition reaction of cyclic nitrones. ChemistrySelect.

[cit31] Acharjee N., Mohammad-Salim H. A., Chakraborty M., Rao M. P., Ganesh M. (2021). Unveiling the high regioselectivity and stereoselectivity within the synthesis of spirooxindolenitropyrrolidine: a molecular electron density theory perspective. J. Phys. Org. Chem..

[cit32] Acharjee N. (2020). Unravelling the regio- and stereoselective synthesis of bicyclic N,O-nucleoside analogues within the molecular electron density theory perspective. Struct. Chem..

[cit33] Domingo L. R., Ríos-Gutiérrez M., Acharjee N. (2019). A molecular electron density theory study of the chemoselectivity, regioselectivity, and diastereofacial selectivity in the synthesis of an anticancer spiroisoxazoline derived from α-Santonin. Molecules.

[cit34] Domingo L. R., Acharjee N. (2021). Unveiling the chemo- and regioselectivity of the [3+2] cycloaddition reaction between 4-chlorobenzonitrile oxide and β-aminocinnamonitrile with a MEDT perspective**. ChemistrySelect.

[cit35] Domingo L. R., Ríos-Gutiérrez M., Silvi B., Pérez P. (2018). The mysticism of pericyclic reactions: a contemporary rationalisation of organic reactivity based on electron density analysis. Eur. J. Org Chem..

[cit36] Mohammad-Salim H. A. (2021). Understanding the reactivity of c-cyclopropyl-n-methylnitrone participating in [3+2] cycloaddition reactions towards styrene with a molecular electron density theory perspective. J. Mex. Chem. Soc..

[cit37] Acharjee N., Mondal A., Chakraborty M. (2022). Unveiling the intramolecular [3 + 2] cycloaddition
reactions of C{,}N-disubstituted nitrones from the molecular electron density theory perspective. New J. Chem..

[cit38] Domingo L., Acharjee N. (2021). Unveiling the substituent effects in the stereochemistry of [3+2] cycloaddition reactions of aryl- and alkyldiazomethylphosphonates with norbornadiene within a MEDT perspective. ChemistrySelect.

[cit39] Domingo L. R., Acharjee N. (2018). [3+2] Cycloaddition reaction of C-phenyl-N-methyl nitrone to acyclic-olefin-bearing electron-donating substituent: a molecular electron density theory study. ChemistrySelect.

[cit40] Domingo L., Ríos-Gutiérrez M., Acharjee N. (2022). A molecular electron density theory study of the lewis acid catalyzed [3+2] cycloaddition reactions of nitrones with nucleophilic ethylenes. Eur. J. Org Chem..

[cit41] Domingo L. R., Ríos-Gutiérrez M., Pérez P. (2018). A molecular electron density theory study of the role of the copper metalation of azomethine ylides in [3 + 2] cycloaddition reactions. J. Org. Chem..

[cit42] Domingo L. R., Acharjee N. (2020). Unravelling the strain-promoted [3+2] cycloaddition reactions of phenyl azide with cycloalkynes from the molecular electron density theory perspective. New J. Chem..

[cit43] Domingo L. R., Acharjee N. (2020). Unveiling the high reactivity of strained dibenzocyclooctyne in [3 + 2] cycloaddition reactions with diazoalkanes through the molecular electron density theory. J. Phys. Org. Chem..

[cit44] Ríos-Gutiérrez M., Domingo L. R. (2019). Unravelling the mysteries of the [3+2] cycloaddition reactions. Eur. J. Org Chem..

[cit45] Domingo L., Acharjee N. (2020). Molecular electron density theory: a new theoretical outlook on organic chemistry. Front. Comput. Chem..

[cit46] Domingo L. R., Ríos-Gutiérrez M. (2017). A molecular electron density theory study of the reactivity of azomethine imine in [3+2] cycloaddition reactions. Molecules.

[cit47] Domingo L. R., Chamorro E., Perez P. (2010). Understanding the high reactivity of the azomethine ylides in [3 + 2] cycloaddition reactions. Lett. Org. Chem..

[cit48] Ríos-Gutiérrez M., Domingo L. R. (2019). The carbenoid-type reactivity of simplest nitrile imine from a molecular electron density theory perspective. Tetrahedron.

[cit49] Jasiński R., Dresler E. (2020). On the question of zwitterionic intermediates in the [3+ 2] cycloaddition reactions: a critical review. Organics.

[cit50] Silvi B., Savin A. (1994). Classification of chemical bonds based on topological analysis of electron localization functions. Nature.

[cit51] Becke A. D., Edgecombe K. E. (1990). A simple measure of electron localization in atomic and molecular systems. J. Chem. Phys..

[cit52] Silvi B. (2002). The synaptic order: a key concept to understand multicenter bonding. J. Mol. Struct..

[cit53] Chellegui M., Benmetir S., Salih R. N., Mohammad-Salim H. A., de Julián-Ortiz J. V., Ben Ahmed A. (2025). A molecular electron density theory investigation of the mechanism of intramolecular [3+2] cycloaddition (32CA) with the participation of nitrile N-oxide and ethene molecular segments. New J. Chem..

[cit54] Ríos-Gutiérrez M., Saz Sousa A., Domingo L. R. (2023). Electrophilicity and nucleophilicity scales at different DFT computational levels. J. Phys. Org. Chem..

[cit55] Domingo L., Ríos-Gutiérrez M., Pérez P. (2016). Applications of the conceptual density functional theory indices to organic chemistry reactivity. Molecules.

[cit56] Parr R. G., Szentpály L. v., Liu S. (1999). Electrophilicity index. J. Am. Chem. Soc..

[cit57] Domingo L. R., Aurell M. J., Pérez P., Contreras R. (2002). Quantitative characterization of the global electrophilicity power of common diene/dienophile pairs in Diels–Alder reactions. Tetrahedron.

[cit58] Makov G. (1995). Chemical hardness in density functional theory. J. Phys. Chem..

[cit59] Domingo L. R., Pérez P. (2011). The nucleophilicity N index in organic chemistry. Org. Biomol. Chem..

[cit60] Zeroual A., Benharref A., El Hajbi A. (2015). Theoretical study of stereoselectivity of the [1 + 2] cycloaddition reaction between (1S,3R,8S)-2,2-dichloro-3,7,7,10 tetramethyltricyclo[6,4,0,01.3]dodec-9-ene and dibromocarbene using density functional theory (DFT) B3LYP/6-31G*(d). J. Mol. Model..

[cit61] Mohammad-Salim H., de Julian-Ortiz J. V. (2023). Theoretical insight into the mechanism and selectivity of the [3+2] cycloaddition reaction of n-methyl-1-phenylmethanimine oxide and bicyclopropylidene with a MEDT perspective. Struct. Chem..

[cit62] Domingo L. R., Chamorro E., Pérez P. (2008). Understanding the reactivity of captodative ethylenes in polar cycloaddition reactions. A theoretical study. J. Org. Chem..

[cit63] Domingo L. R., Aurell M. J., Pérez P., Contreras R. (2002). Quantitative characterization of the local electrophilicity of organic molecules. understanding the regioselectivity on diels−alder reactions. J. Phys. Chem. A.

[cit64] Mondal A., Mohammad-Salim H. A., Acharjee N. (2023). Unveiling substituent effects in [3+2] cycloaddition reactions of benzonitrile N-oxide and benzylideneanilines from the molecular electron density theory perspective. Sci. Radices.

[cit65] Adjieufack A. I., Andrés J., Oliva M., Safont V. S. (2022). Deciphering the molecular mechanism of intramolecular reactions from the perspective of bonding evolution theory. Physchem.

[cit66] Adjieufack A. I., Moto Ongagna J., Pouyewo Tenambo A., Opoku E., Mbouombouo I. N. (2022). How a chromium tricarbonyl complex catalyzes the [3 + 2] cycloaddition reaction of n-substituted phenylnitrones with styrene: a molecular electron density theory analysis. Organometallics.

[cit67] Aitbraim I., Baammi S., ouled aitouna A., Zeroual A., Chekroun A., Mohammad-Salim H., Al-Sadoon M., Belghiti M. E., de Julian-Ortiz J. V., Benharref A. (2024). Quantum evaluation of novel epoxides: molecular docking, dynamics simulation, pharmacokinetics, stereoselectivity, and mechanistic insights into cis-himachalone and cis-himachalol epoxidation. Chem. Heterocycl. Compd..

[cit68] Banerjee B., Domingo L. R., Mohammad-Salim H. A., Mondal A., Acharjee N. (2025). Understanding [3+2] cycloaddition reactions of difluoroallene to nitrone and diazoalkanes from the molecular electron density theory perspective. New J. Chem..

[cit69] Adjieufack A. I., Nana C. N., Ketcha-Mbadcam J., Mbouombouo Ndassa I., Andrés J., Oliva M., Safont V. S. (2020). Deciphering the curly arrow representation and electron flow for the 1,3-dipolar rearrangement between acetonitrile oxide and (1s,2r,4s)-2-cyano-7-oxabicyclo[2.2.1]hept-5-en-2-yl acetate derivatives. ACS Omega.

[cit70] Adjieufack A. I., Liégeois V., Ndassa Mboumbouo I., Ketcha Mbadcam J., Champagne B. (2018). Intramolecular [3 + 2] cycloaddition reactions of unsaturated nitrile oxides. A study from the perspective of bond evolution theory (BET). J. Phys. Chem. A.

[cit71] Cyrille N. N., Idrice A. A., Maraf M. B., Charles F. A., Ibrahim M. N., Ríos-Gutierrez M., Domingo L. R. (2019). Understanding the mechanism of nitrobenzene nitration with nitronium ion: a molecular electron density theory study. ChemistrySelect.

[cit72] Cherni E., Adjieufack A. I., Champagne B., Abderrabba M., Ayadi S., Liégeois V. (2020). Density functional theory investigation of the binding of ThioTEPA to purine bases: thermodynamics and bond evolution theory analysis. J. Phys. Chem. A.

[cit73] Hellel D., Chafaa F., Khorief Nacereddine A., Djerourou A., Vrancken E. (2017). Regio- and stereoselective synthesis of novel isoxazolidine heterocycles by 1,3-dipolar cycloaddition between *C*-phenyl-*N*-methylnitrone and substituted alkenes. Experimental and DFT investigation of selectivity and mechanism. RSC Adv..

[cit74] Li C., Wang C., Villa-Marcos B., Xiao J. (2008). Chiral counteranion-aided asymmetric hydrogenation of acyclic imines. J. Am. Chem. Soc..

[cit75] Salih S., Basheer H., Mohammad-Salim H. (2022). Unveiling [3 + 2] cycloaddition reactions of *N*-methyl-C-3-bromophenyl-nitrone to dimethyl maleate: molecular electron density theory perspective. J. Mex. Chem. Soc..

[cit76] Benmetir S., Benhamed L., Tchouar N., Mohammad-Salim H., Vicente de Julián-Ortiz J., Ríos-Gutiérrez M., Domingo L. R. (2025). Unveiling hydrogen bonding and solvent effects on directed nitrile oxide [3 + 2] cycloaddition reactions: selectivity of 2,2-dimethylpropane nitrile oxide with cyclopentenylbenzamide: an MEDT study. ACS Omega.

[cit77] Nacereddine A. K., Sobhi C., Djerourou A., Ríos-Gutiérrez M., Domingo L. R. (2015). Non-classical CH⋯O hydrogen-bond determining the regio- and stereoselectivity in the [3 + 2] cycloaddition reaction of (Z)-C-phenyl-*N*-methylnitrone with dimethyl 2-benzylidenecyclopropane-1{,}1-dicarboxylate. A topological electron-density study. RSC Adv..

[cit78] Polo V., Gonzalez-Navarrete P., Silvi B., Andres J. (2008). An electron localization function and catastrophe theory analysis on the molecular mechanism of gas-phase identity SN2 reactions. Theor. Chem. Acc..

[cit79] Khorief Nacereddine A. (2020). A MEDT computational study of the mechanism, reactivity and selectivity of non-polar [3+2] cycloaddition between quinazoline-3-oxide and methyl 3-methoxyacrylate. J. Mol. Model..

[cit80] Salih R. N., Mohammad-Salim H., Algso M. (2025). Computational investigation of the cycloaddition reaction mechanism of 2,4,6-trimethyl-3,5-dichlorobenzonitrile *N*-oxide with arylacetylenes: insights from density functional theory and molecular docking. React. Kinet., Mech. Catal..

[cit81] Mohammad-Salim H., Hassan R., Abdallah H. H., Oftadeh M. (2020). Theoretical study on the mechanism of [3+2] cycloaddition reactions between α,β-unsaturated selenoaldehyde with nitrone and with nitrile oxide. J. Mex. Chem. Soc..

[cit82] KUMAR P. S. V., RAGHAVENDRA V., SUBRAMANIAN V. (2016). Bader's theory of atoms in molecules (AIM) and its applications to chemical bonding. J. Chem. Sci..

[cit83] Mohammad-Salim H. A., Acharjee N., Domingo L. R., Abdallah H. H. (2021). A molecular electron density theory study for [3+2] cycloaddition reactions of 1-pyrroline-1-oxide with disubstituted acetylenes leading to bicyclic 4-isoxazolines. Int. J. Quantum Chem..

[cit84] Kącka-Zych A. (2020). Push-pull nitronates in the [3+ 2] cycloaddition with nitroethylene: molecular electron density theory study. J. Mol. Graphics Modell..

[cit85] Hallooman D., Rios-Gutierrez M., Rhyman L., Alswaidan I. A., Domingo L. R., Ramasami P. (2018). DFT exploration of [3+ 2] cycloaddition reaction of 1 H-phosphorinium-3-olate and 1-methylphosphorinium-3-olate with methyl methacrylate. RSC Adv..

[cit86] Pipim G. B., Tia R., Adei E. (2021). Quantum chemical investigation of the formation of spiroheterocyclic compounds *via* the (3+2) cycloaddition reaction of 1-methyl-3-(2,2,2-trifluoroethylidene) pyrrolidin-2-one with diazomethane and nitrone derivatives. Tetrahedron.

[cit87] Tomasi J., Mennucci B., Cammi R. (2005). Quantum mechanical continuum solvation models. Chem. Rev..

[cit88] Wolters L. P., Bickelhaupt F. M. (2015). The activation strain model and molecular orbital theory. Wiley Interdiscip. Rev.:Comput. Mol. Sci..

[cit89] Reed A. E., Curtiss L. A., Weinhold F. (1988). Intermolecular interactions from a natural bond orbital, donor-acceptor viewpoint. Chem. Rev..

[cit90] Fukui K. (1970). Formulation of the reaction coordinate. J. Phys. Chem..

[cit91] Gonzalez C., Schlegel H. B. (1990). Reaction path following in mass-weighted internal coordinates. J. Phys. Chem..

[cit92] Gonzalez C., Schlegel H. B. (1991). Improved algorithms for reaction path following: Higher-order implicit algorithms. J. Chem. Phys..

[cit93] Noury S., Krokidis X., Fuster F., Silvi B. (1999). Computational tools for the electron localization function topological analysis. Comput. Chem..

[cit94] Lu T., Chen F. (2012). Multiwfn: a multifunctional wavefunction analyzer. J. Comput. Chem..

[cit95] Liégeois V. , DrawMol, UNamur, 2024

[cit96] Liégeois V. , DrawProfile, UNamur, 2024

[cit97] Frisch M. , Trucks G. W., Schlegel H. B., Scuseria G. E., Robb M. A., Cheeseman J. R., Scalmani G., Barone V., Mennucci B., Petersson G. A., *et al.*, Gaussian 16, Revision A. 03, Gaussian Inc., Wallingford, CT, 2016

[cit98] Ríos-Gutiérrez M., Domingo L. (2018). Unravelling the mysteries of the [3+2] cycloaddition reactions. Eur. J. Org Chem..

[cit99] R Domingo L., Chamorro E., Pérez P. (2010). Understanding the high reactivity of the azomethine ylides in [3+ 2] cycloaddition reactions. Lett. Org. Chem..

[cit100] Chellegui M., Trabelsi M., Champagne B., Liegeois V. (2025). DFT Investigation of the stereoselectivity of the lewis-acid-catalyzed diels–alder reaction between 2,5-dimethylfuran and acrolein. ACS Omega.

[cit101] Chellegui M., Adjieufack A. I., Trabelsi M., Liégeois V., Champagne B. (2025). Unveiling the reaction mechanism of diels-alder cycloadditions between 2, 5-dimethylfuran and ethylene derivatives using topological tools. ChemPhysChem.

[cit102] Chellegui M., Koudjina S., Salhi I., Benmetir S., Salih R. N., Mohammad-Salim H. A., de Julián-Ortiz J. V. (2025). Mechanism and stereoselectivity of a [3+ 2] cycloaddition involving a glucosyl nitrone: a MEDT study. Org. Biomol. Chem..

[cit103] Benmetir S., Chellegui M., Benhamed L., Al-Mokhtar R. M., Salih R. N., Algso M. A., Mohammad-Salim H. A. (2025). Mechanistic insights into the regio-and stereoselectivity of [3+ 2] cycloaddition reactions between N-methyl-phenylnitrone and trans-1-chloro-2-nitroethylene within the framework of molecular electron density theory. New J. Chem..

[cit104] Chellegui M., Salhi I., Ben Ahmed A., Benmetir S., Salih R. N., Mohammad-Salim H. A., de Julián-Ortiz J. V. (2025). Mechanistic study of the [2+ 2] cycloaddition of ethylene with ketene derivatives *via* MEDT. Struct. Chem..

[cit105] Chellegui M., Champagne B., Trabelsi M. (2022). Lewis acid-catalyzed Diels–Alder cycloaddition of 2,5-dimethylfuran and ethylene: a density functional theory investigation. Theor. Chem. Acc..

[cit106] Domingo L. R., Ríos-Gutiérrez M., Pérez P. (2025). Electrophilicity ω and nucleophilicity N scales for cationic and anionic species. Sci. Rad..

[cit107] Domingo L. R., Ríos-Gutiérrez M., Pérez P. (2020). RSC Adv..

[cit108] Jasiński R. (2015). A stepwise, zwitterionic mechanism for the 1,3-dipolar cycloaddition between (*Z*)-C-4-methoxyphenyl-*N*-phenylnitrone and gem-chloronitroethene catalysed by 1-butyl-3-methylimidazolium ionic liquid cations. Tetrahedron Lett..

[cit109] Domingo L. R., Ríos-Gutiérrez M. (2023). A useful classification of organic reactions based on the flux of the electron density. Sci. Radices.

