# Supplementary material for: Mechanistic insights into the (3 + 2) cycloaddition of azomethine ylide with dimethyl acetylenedicarboxylate via bond evolution theory
Source: RSC Adv. 2025 Aug 21;15(36):29666–79. doi: 10.1039/d5ra04992j (PMC12376994; doi:10.1039/d5ra04992j)
Supplement: RA-015-D5RA04992J-s001 [file RA-015-D5RA04992J-s001.pdf]

Supplementary Information (SI) for RSC Advances.
This journal is © The Royal Society of Chemistry 2025

"Supporting Information"

Mechanistic Insights Into The [3+2] Cycloaddition Of Azomethine Ylide With Dimethyl Acetylenedicarboxylate Via Bond Evolution Theory

Mohamed Chellegui[a,b], Raghad Mowafak Al-Mokhtar[c], Raad Nasrullah Salih[d], Lakhdar Benhamed[e], Sofiane Benmetir[f,g], Jesus Vicente de Julián-Ortiz[f], Haydar A. Mohammad-Salim[h,i], and Ali Ben Ahmed*[j,k]

[a]Laboratory of Organic Chemistry (LR17ES08), Faculty of Sciences, University of Sfax, 3038 Sfax, Tunisia.
[b]Namur Institute of Structured Matter, University of Namur, Rue de Bruxelles, 61, B-5000 Namur, Belgium.
[c]Department of Chemistry, College of Science, University of Duhok, Duhok 42001, Kurdistan Region, Iraq.
[d]Nursing Department, Bardarash Technical Institute, Akre University for Applied Science, Duhok 42001, Kurdistan Region, Iraq
[e]Laboratory of Applied Thermodynamics and Molecular Modelling (LAT2M), Department of Chemistry, Faculty of Science, University of Tlemcen, PB 119, Tlemcen, 13000, Algeria.
[f]Department of Physical Chemistry, Faculty of Pharmacy, University of Valencia, Av. Vicente Andrés Estellés s/n, 46100 Valencia, Spain.
[g]Process and Environmental Engineering Laboratory (LIPE), Faculty of Chemistry, University of Science and Technology of Oran Mohamed BOUDIAF, P.O. Box 1503, El Mnaouer, 31000 Oran, Algeria.
[h]Department of Chemistry, Faculty of Science, University of Zakho, Zakho 42002, Kurdistan Region, Iraq.
[i]TCCG Lab, Scientific Research Center, University of Zakho, Zakho 42002, Kurdistan Region, Iraq.
[j]Laboratory of Applied Physics, Department of Physics, Faculty of Sciences of Sfax, University of Sfax, Sfax-Tunisia.
[k]Department of Biomedical, Higher Institute of Biotechnology of Sfax, University of Sfax, Sfax-Tunisia.

*Corresponding author: ali.benahmed@isbs.usf.tn

In this work, DFT at the M06-2X-D3/6–311G(d,p) level of approximation, in benzene at ambient temperature, is employed to describe the 32CA reaction between azomethine ylides **1** and dimethyl acetylenedicarboxylate **2** (**Scheme S1**).

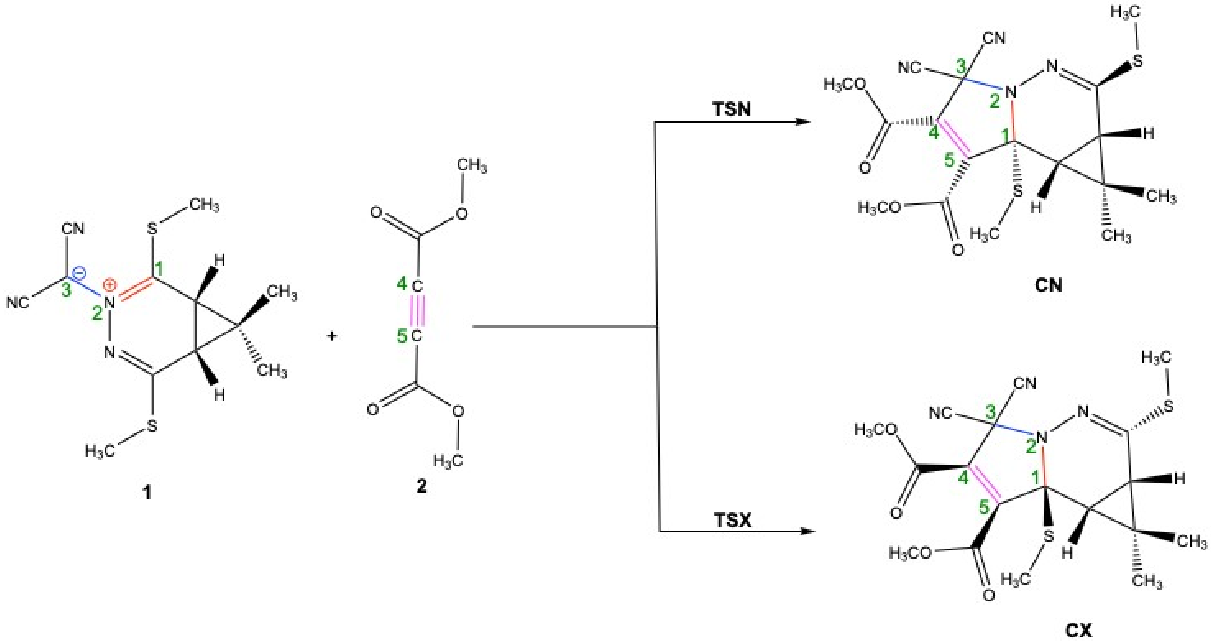

Scheme S1 32CA reactions stereoisomeric paths between azomethine ylides **1** and dimethyl acetylenedicarboxylate **2**. [**CN** = endo cycloadduct, **CX** = exo cycloadduct, **TSN** = TS-endo, and **TSX** = TS-exo].

Table S1. Local reactivity descriptors—including nucleophilic (N_k) and electrophilic (ω_k) indices, as well as the local reactivity difference index (R_k) were calculated at the IEFPCM(1,4-dioxane)/B3LYP-D3/6-311++G(d, p) level. Here, *k* denotes each reactive center in reactants **1** and **2**.

**Reactants**	k	P_k^-	N_k	P_k^+	ω_k	R_k

**1**	C1	0.06	0.22	0.004	0.01	-0.21
	N2	-0.06	-0.22	0.001	0.003	0.22
	C3	0.70	2.59	-0.01	-0.03	-2.59
**2**	C4=C5	0.15	0.14	0.24	0.50	0.36

Table S2. Energies, enthalpies, entropies, and Gibbs enthalpies of reactants, transition states, and products calculated at the IEFPCM(1,4-dioxane)/B3LYP-D3/6-311++G(d,p) level of approximation.

	**E°**	**H°**	**S°**	**G°**
**1**	-1481.235311	-1480.973095	145.122	-1481.040891
**2**	-533.252589	-533.126757	105.525	-533.176054
**CX**	-2014.562689	-2014.171341	193.301	-2014.261644
**CN**	-2014.569591	-2014.178107	197.563	-2014.270402
**TSX**	-2014.476684	-2014.088326	199.698	-2014.181618
**TSN**	-2014.472528	-2014.084633	200.349	-2014.178229

Table S3 Basin populations (in e), IRC coordinates (RX, Bohr AMU$^{1/2}$), relative electronic energies (ΔE in kcal/mol) and C1-C4/O3-C5 bond lengths (in Å) along the **TSX** stereoisomeric channel of the studied 32CA reaction.

	**SSD-I**		**SSD-II**		**SSD-III**		**SSD-IV**		**SSD-V**		**SSD-VI**	
**Basins**	First	Last	First	Last	First	Last	First	Last	First	Last	First	Last
**V(C1,N2)**	3.78	3.80	3.04	2.91	2.86	2.65	2.59	2.46	2.43	2.11	2.06	1.95
**V(C4,C5)**	5.27	5.07	5.04	4.98	4.91	4.55	4.47	4.30	4.22	3.80	3.78	3.50
**V(C3,N2)**	2.10	2.07	2.04	2.03	2.02	1.94	1.92	1.89	1.88	1.86	1.78	1.73
**V(C3)**	0.63	0.79	0.81	0.90	0.92	0.98	0.99	1.04	---	---	---	---
**V(N2)**	---	---	0.79	0.96	1.04	1.27	1.36	1.55	1.59	2.05	2.10	2.20
**V(C1)**	---	---	---	---	---	---	---	---	0.29	0.59	---	---
**V(C4)**	---	---	---	---	0.14	0.35	0.40	0.49	---	---	---	---
**V(C5)**	---	---	---	---	---	---	0.56	0.69	0.75	1.04	---	---
**V(C1,C5)**	---	---	---	---	---	---	---	---	---	---	1.69	2.13
**V(C3,C4)**	---	---	---	---	---	---	---	---	1.59	1.84	1.88	1.99
**d(C1,C5)**	3.367	2.791	2.767	2.664	2.636	2.541	2.505	2.434	2.397	2.041	1.978	1.520
**d(C3,C5)**	3.47	2.607	2.561	2.513	2.363	2.311	2.154	2.100	1.990	1.664	1.646	1.541
**RX**	-16.44	-3.42	-3.08	-1.71	-1.37	-0.34	0.00	0.69	1.03	3.42	3.77	34.24
**E**	38.87	48.57	49.14	51.51	52.06	53.20	53.31	52.75	51.96	39.04	36.48	0.00

Cartesian Coordinates of all the Stationary Points as Computed at the IEFPCM(1,4-dioxane)/B3LYP-D3/6-311++G(d,p) level of approximation

1

0 1

C	1.7401830000	-0.2979020000	-0.2609260000
C	1.1438940000	-1.6366100000	-0.4269460000
C	-0.3870470000	-1.6223310000	-0.5616460000
C	-1.0243240000	-0.3083510000	-0.4781770000
H	1.6927580000	-2.3193420000	-1.0656530000
H	-0.8163650000	-2.3089700000	-1.2754960000
C	0.2863380000	-2.2483940000	0.6600260000
C	0.2628770000	-3.7667400000	0.6930250000
H	1.1002390000	-4.1404060000	1.2885470000
H	-0.6642170000	-4.1196590000	1.1527320000
H	0.3369610000	-4.2010640000	-0.3068430000
C	0.1696160000	-1.6065530000	2.0278370000
H	0.9757280000	-1.9744400000	2.6677980000
H	0.2290890000	-0.5201040000	2.0153310000
H	-0.7824390000	-1.8844280000	2.4864260000
N	1.0857960000	0.7879290000	-0.0468930000
N	-0.3176360000	0.7429060000	-0.0744820000
C	-0.9400700000	1.9666360000	0.3002800000
C	-0.4186630000	3.1605420000	-0.2131410000
N	0.0033760000	4.1521820000	-0.6500350000
C	-1.8664080000	1.9422160000	1.3473630000
N	-2.6518990000	1.8869480000	2.2051800000
S	3.4920740000	-0.2334280000	-0.3715770000
S	-2.7115000000	-0.0416420000	-0.8257850000
C	-3.2422600000	-1.6448340000	-1.5364410000
H	-3.1247260000	-2.4548070000	-0.8177760000
H	-4.3042100000	-1.5023950000	-1.7376730000
H	-2.7293670000	-1.8641360000	-2.4720750000
C	3.7993320000	1.5620610000	-0.2432510000
H	3.4654770000	1.9306740000	0.7246730000
H	4.8772330000	1.6839880000	-0.3467870000
H	3.2763770000	2.0880200000	-1.0398190000

2

0 1

C	-0.5988360000	0.1904030000	-0.0623900000
C	0.5988360000	0.1903920000	0.0624210000
C	2.0285730000	0.2480600000	0.2820420000
C	-2.0285730000	0.2481050000	-0.2820020000
O	-2.5467430000	0.9756120000	-1.0958330000
O	2.5467430000	0.9754390000	1.0959860000
O	2.6750970000	-0.5986850000	-0.5257660000
O	-2.6750980000	-0.5987660000	0.5256730000
C	-4.1178630000	-0.6222800000	0.3931440000
H	-4.3958890000	-0.9160200000	-0.6194630000
H	-4.4568990000	-1.3580030000	1.1173550000
H	-4.5300520000	0.3621460000	0.6166250000
C	4.1178630000	-0.6222170000	-0.3932440000
H	4.4568990000	-1.3578250000	-1.1175720000
H	4.5300500000	0.3622450000	-0.6165710000
H	4.3958920000	-0.9161170000	0.6193160000

CX

0 1

C	-1.5626730000	0.5565420000	-0.1030350000
C	-1.0059260000	-0.6347100000	-0.3354300000
C	-1.6087080000	-1.7168370000	-1.1769460000
C	-2.8926670000	1.0025290000	-0.5773700000

O	-3.5141970000	0.4622720000	-1.4633900000
O	-1.1355730000	-2.0525570000	-2.2366260000
O	-2.6705320000	-2.2650780000	-0.5928320000
O	-3.2977600000	2.0806940000	0.1017040000
C	-4.5740010000	2.6465830000	-0.2750390000
H	-5.3660270000	1.9128980000	-0.1228110000
H	-4.7090490000	3.5048010000	0.3773900000
H	-4.5497620000	2.9527950000	-1.3211950000
C	-3.3802840000	-3.2625900000	-1.3678640000
H	-4.1890620000	-3.5997310000	-0.7254100000
H	-3.7704540000	-2.8060150000	-2.2775510000
H	-2.7139710000	-4.0874260000	-1.6227470000
C	2.4868620000	0.8292480000	-0.6050870000
C	2.5213420000	-0.5908740000	-0.9963170000
C	1.4115600000	-1.4503610000	-0.4296260000
C	0.3247930000	-0.7836160000	0.3756970000
H	2.8458920000	-0.7842810000	-2.0123700000
H	1.0282230000	-2.2065520000	-1.1010190000
C	2.8282250000	-1.6535660000	0.0568010000
C	3.5290470000	-2.8997420000	-0.4556250000
H	4.6104800000	-2.7395730000	-0.4906790000
H	3.3371460000	-3.7462110000	0.2109750000
H	3.1938910000	-3.1743290000	-1.4589770000
C	3.2919960000	-1.2243170000	1.4363560000
H	4.3582090000	-0.9822400000	1.3970970000
H	2.7592760000	-0.3574550000	1.8226090000
H	3.1509030000	-2.0396690000	2.1489510000
N	1.6381110000	1.3690120000	0.1836970000
N	0.6402470000	0.6102000000	0.8056900000
C	-0.6343270000	1.3879840000	0.7980530000
C	-1.1810940000	1.4914330000	2.1727430000
N	-1.6067190000	1.5708370000	3.2393220000
C	-0.4443170000	2.7559940000	0.2730050000
N	-0.4048020000	3.8288760000	-0.1419200000
S	3.7569280000	1.8350900000	-1.3224540000
S	-0.0214330000	-1.7228400000	1.9858160000
C	-0.1149240000	-3.4503610000	1.3959940000
H	0.8068790000	-3.7423970000	0.8937570000
H	-0.2318270000	-4.0535410000	2.2973090000
H	-0.9774170000	-3.6158670000	0.7520840000
C	3.3292420000	3.4808040000	-0.6596800000
H	3.4007200000	3.4780880000	0.4268470000
H	4.0583530000	4.1707700000	-1.0846170000
H	2.3214300000	3.7599590000	-0.9615910000

CN
0 1

C	1.2503080000	-0.6837360000	0.0520350000
C	1.6572820000	0.5766220000	-0.1301550000
C	3.0290720000	1.0197270000	-0.4708870000
C	2.0585620000	-1.9343880000	-0.0841460000
O	1.8171310000	-2.7507810000	-0.9410680000
O	3.9028810000	0.2830150000	-0.8626710000
O	3.1512580000	2.3397770000	-0.2919490000
O	3.0006850000	-2.0288080000	0.8451100000
C	3.8643720000	-3.1888600000	0.7635620000
H	4.4108540000	-3.1728090000	-0.1796810000
H	4.5436600000	-3.0978150000	1.6066090000
H	3.2734100000	-4.1022060000	0.8361080000
C	4.4364880000	2.9233590000	-0.6088490000
H	4.3261370000	3.9845410000	-0.4037710000
H	5.2098740000	2.4829070000	0.0208200000
H	4.6725700000	2.7519080000	-1.6593770000

C	-2.8218150000	0.1429450000	-0.1267810000
C	-2.5509480000	-1.2900850000	-0.4036760000
C	-1.1179370000	-1.7709930000	-0.1670080000
C	-0.2116940000	-0.7264700000	0.4297570000
H	-3.3585980000	-1.9641960000	-0.1409870000
H	-0.9948620000	-2.7547460000	0.2688880000
C	-1.6562550000	-1.7234820000	-1.5740240000
C	-1.9888290000	-3.0892880000	-2.1549260000
H	-2.7814230000	-3.0025880000	-2.9034330000
H	-1.1032100000	-3.5105810000	-2.6388370000
H	-2.3210570000	-3.7929470000	-1.3874300000
C	-1.1814600000	-0.7421690000	-2.6302450000
H	-1.8551580000	-0.7841200000	-3.4902670000
H	-1.1459640000	0.2877910000	-2.2881640000
H	-0.1824930000	-1.0272610000	-2.9722690000
N	-1.9270760000	1.0444060000	0.0878000000
N	-0.6290510000	0.5908570000	0.0119090000
C	0.4588580000	1.5386470000	0.0247810000
C	0.5518660000	2.3489220000	1.2723670000
N	0.5869300000	2.9765300000	2.2371120000
C	0.3331780000	2.4574550000	-1.1300630000
N	0.2224450000	3.1364410000	-2.0527300000
S	-4.5319100000	0.5787550000	-0.0657930000
S	-0.1178080000	-0.9623190000	2.3353380000
C	-1.8693090000	-1.1636520000	2.8286510000
H	-2.3275240000	-2.0206770000	2.3375330000
H	-1.8389170000	-1.3471250000	3.9037540000
H	-2.4416120000	-0.2587290000	2.6342740000
C	-4.4331900000	2.3648860000	0.2901660000
H	-3.9141030000	2.8810470000	-0.5157820000
H	-5.4636800000	2.7128780000	0.3601490000
H	-3.9120710000	2.5318550000	1.2317920000

TSX

0 1

C	1.8485560000	0.4863350000	-0.6080290000
C	0.8304610000	1.0362940000	-1.0771870000
C	-0.0834300000	1.6966560000	-1.9351140000
C	3.2879770000	0.3422320000	-0.8780700000
O	3.7744240000	0.4684400000	-1.9780280000
O	-0.7306260000	1.1561900000	-2.8145470000
O	-0.2127030000	3.0159780000	-1.6222010000
O	3.9834650000	0.0739230000	0.2336130000
C	5.4043970000	-0.1201330000	0.0740950000
H	5.8631040000	0.7770980000	-0.3431830000
H	5.7797350000	-0.3174830000	1.0748950000
H	5.5925740000	-0.9693670000	-0.5838570000
C	-1.0980800000	3.7712920000	-2.4742260000
H	-1.0596260000	4.7909060000	-2.0967740000
H	-0.7563210000	3.7349950000	-3.5092080000
H	-2.1157980000	3.3795710000	-2.4202390000
C	-1.5915180000	-1.7475540000	-0.4632280000
C	-2.5765530000	-0.6549640000	-0.4604520000
C	-2.0917480000	0.5955960000	0.2494720000
C	-0.7360660000	0.5702500000	0.8215220000
H	-3.1336490000	-0.5254580000	-1.3802080000
H	-2.3538480000	1.5379780000	-0.2091480000
C	-3.2392560000	-0.2160010000	0.8357170000
C	-4.6169850000	0.4041490000	0.6872310000
H	-5.3829300000	-0.3761980000	0.6830230000
H	-4.8229760000	1.0751490000	1.5262270000
H	-4.7108210000	0.9763370000	-0.2387410000
C	-3.0552040000	-1.0064450000	2.1149950000

H	-3.7984720000	-1.8066930000	2.1605780000
H	-2.0706530000	-1.4617200000	2.2097230000
H	-3.2069810000	-0.3532000000	2.9783390000
N	-0.4709940000	-1.7445280000	0.1642980000
N	-0.0715570000	-0.6060560000	0.8832210000
C	1.3443010000	-0.6608690000	1.0765230000
C	1.8976680000	-0.0750050000	2.2652810000
N	2.4002680000	0.3445130000	3.2180480000
C	1.9295870000	-1.9468810000	0.8059930000
N	2.5163720000	-2.9186420000	0.5903190000
S	-2.0125650000	-3.1521700000	-1.4222600000
S	-0.2355660000	1.9005160000	1.8716550000
C	-1.5030650000	3.1759140000	1.5498560000
H	-2.5028220000	2.8198670000	1.7920970000
H	-1.2330150000	3.9834800000	2.2309820000
H	-1.4336280000	3.5335070000	0.5248200000
C	-0.4867580000	-4.1471780000	-1.2974500000
H	-0.3072720000	-4.4376890000	-0.2642980000
H	-0.6565960000	-5.0266940000	-1.9179490000
H	0.3592740000	-3.5732970000	-1.6708900000

TSN

0 1

C	1.5970210000	-0.6687630000	-0.5602000000
C	1.7499890000	0.5505720000	-0.7022760000
C	2.2843250000	1.7329060000	-1.3622690000
C	1.9629040000	-2.0537020000	-0.7788660000
O	1.3158090000	-2.8644810000	-1.4066990000
O	2.1985550000	1.9155570000	-2.5557970000
O	2.8481660000	2.5765010000	-0.4927320000
O	3.1226020000	-2.3321070000	-0.1580630000
C	3.5772060000	-3.7001440000	-0.2593780000
H	3.7452050000	-3.9675240000	-1.3032700000
H	4.5077770000	-3.7329680000	0.3016330000
H	2.8391740000	-4.3751420000	0.1764340000
C	3.3264140000	3.8288780000	-1.0357300000
H	3.7494560000	4.3612980000	-0.1880060000
H	4.0841280000	3.6454680000	-1.7980680000
H	2.4929990000	4.3857230000	-1.4650570000
C	-2.8036610000	0.0575280000	-0.0157380000
C	-2.6519010000	-1.3976680000	-0.0264010000
C	-1.2334220000	-1.8655850000	0.3665900000
C	-0.2972900000	-0.8416120000	0.8628250000
H	-3.5064880000	-1.9834420000	0.2920600000
H	-1.1733870000	-2.7979420000	0.9110320000
C	-1.7141620000	-1.9862310000	-1.0683690000
C	-2.0353600000	-3.4045010000	-1.5059040000
H	-2.7607740000	-3.3899240000	-2.3241710000
H	-1.1205860000	-3.8861820000	-1.8571560000
H	-2.4528420000	-4.0027510000	-0.6919660000
C	-1.1943660000	-1.1153080000	-2.1948030000
H	-1.9808640000	-1.0010130000	-2.9462230000
H	-0.8829300000	-0.1221610000	-1.8812280000
H	-0.3353530000	-1.6005890000	-2.6555700000
N	-1.8663710000	0.9122500000	0.2122410000
N	-0.6382620000	0.4650200000	0.7193800000
C	0.2790340000	1.5237290000	0.8058260000
C	1.1764160000	1.6725620000	1.9046030000
N	1.8958750000	1.9402860000	2.7702910000
C	-0.1417490000	2.7676530000	0.2501720000
N	-0.3538940000	3.8049960000	-0.2163250000
S	-4.3993260000	0.6596290000	-0.4292730000
S	0.7685630000	-1.3272520000	2.2246070000

C	-0.3863780000	-1.0064360000	3.6177690000
H	-1.2950790000	-1.6003360000	3.5194350000
H	0.1494320000	-1.3052870000	4.5197230000
H	-0.6203480000	0.0562300000	3.6775120000
C	-4.1712970000	2.4639910000	-0.2640190000
H	-3.4157010000	2.8125090000	-0.9649890000
H	-5.1405120000	2.9043890000	-0.4970220000
H	-3.8759210000	2.7127720000	0.7537680000
